# Recent Clinical and Preclinical Studies of Hydroxychloroquine on RNA Viruses and Chronic Diseases: A Systematic Review

**DOI:** 10.3390/molecules25225318

**Published:** 2020-11-14

**Authors:** Immacolata Faraone, Fabiana Labanca, Maria Ponticelli, Nunziatina De Tommasi, Luigi Milella

**Affiliations:** 1 Department of Science, University of Basilicata, v.le dell’Ateneo Lucano 10, 85100 Potenza, Italy; immacolata.faraone@unibas.it (I.F.); fabiana.labanca@unibas.it (F.L.); maria.ponticelli@unibas.it (M.P.); luigi.milella@unibas.it (L.M.); 2 Spinoff BioActiPlant s.r.l., University of Basilicata, v.le dell’Ateneo Lucano 10, 85100 Potenza, Italy; 3 Department of Pharmacy, Università degli Studi di Salerno, Via Giovanni Paolo II, 132, 84084 Fisciano, Italy

**Keywords:** hydroxychloroquine, structure-activity relationship, antiviral, mechanism of action, biological activity, synergistic effects, toxicological effects, preclinical study, clinical study, animal model

## Abstract

The rapid spread of the new Coronavirus Disease 2019 (COVID-19) has actually become the newest challenge for the healthcare system since, to date, there is not an effective treatment. Among all drugs tested, Hydroxychloroquine (HCQ) has attracted significant attention. This systematic review aims to analyze preclinical and clinical studies on HCQ potential use in viral infection and chronic diseases. A systematic search of Scopus and PubMed databases was performed to identify clinical and preclinical studies on this argument; 2463 papers were identified and 133 studies were included. Regarding HCQ activity against COVID-19, it was noticed that despite the first data were promising, the latest outcomes highlighted the ineffectiveness of HCQ in the treatment of viral infection. Several trials have seen that HCQ administration did not improve severe illness and did not prevent the infection outbreak after virus exposure. By contrast, HCQ arises as a first-line treatment in managing autoimmune diseases such as rheumatoid arthritis, lupus erythematosus, and Sjögren syndrome. It also improves glucose and lipid homeostasis and reveals significant antibacterial activity.

## 1. Introduction

The 4-aminoquinolonehydroxychloroquine (HCQ) was synthesized for the first time in 1946, but its history began as far back as the 1600s thanks to the Incas in Chile. They introduced the special properties of cinchona bark to the Jesuits and in 1820 quinine and cinchonine were isolated and identified as the main alkaloids responsible for the antimalarial activity attributed to the bark. For these reasons, in 1900, the Dutch and British transplanted this “miraculous tree” to Java. The quinine soon began to be also used for the treatment of systemic lupus erythematosus [1,2,3].

The increasing need for quinine, due to malaria diffusion, led the pharmaceutical industry to the creation of a synthetic molecule. In 1934 Johann (Hans) Andersag and co-workers at the Bayer I.G. Farbenindustrie in Elberfeld laboratories, Germany, synthesized chloroquine (CQ) for the first time, judged by the Germans to be too toxic [4]. 

**Figure 1:**
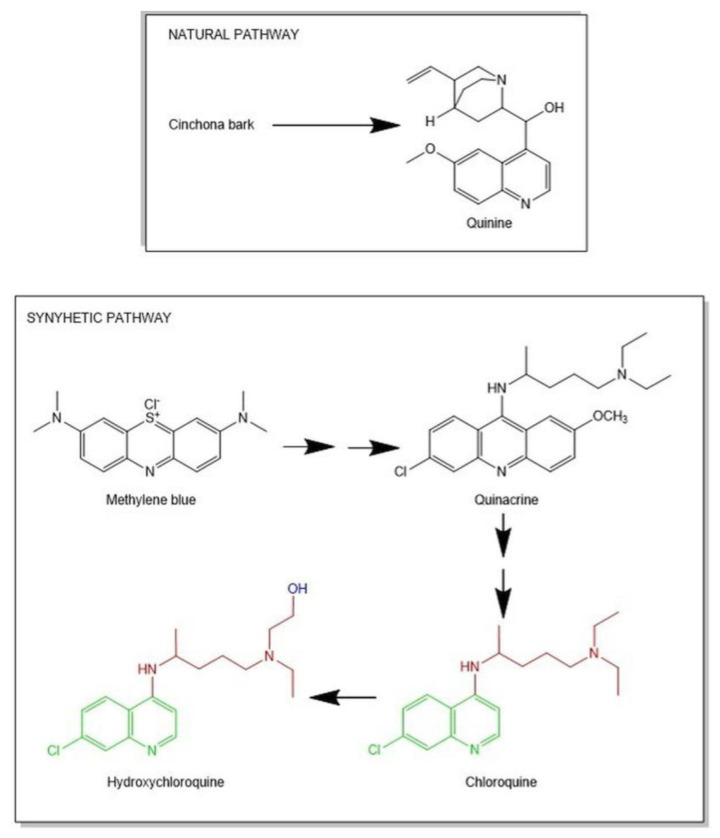
Historical development of hydroxychloroquine (HCQ) synthesis. In green is represented the 4-aminoquinoline nucleus, in red is the amphiphilic weak basic side chain and in blue, the hydroxyl group.

However, the production of natural quinine was blocked by the Japanese army’s occupation of Java during the Second World War. Therefore, it was necessary to deepen the studies on molecules that could replace natural quinine. The Americans soon synthesized from CQ the HCQ, which resulted in it being less toxic than its ancestor so that in 1955 the scientific community proposed it as an alternative to CQ. Figure 1 represents the historical development that led to HCQ synthesis [5].

This molecule, which has always been known as antimalarial, has come back into vogue in recent months due to the ongoing new coronavirus (2019-nCoV, or COVID-19 or 2019-nCo) worldwide pandemic, that causes a severe acute respiratory syndrome (SARS-CoV-2). In fact, several research groups have evaluated the use of CQ (C_18_H_26_ClN_3_) and in particular its derivative HCQ (C_18_H_26_ClN_3_O), as a promising treatment for COVID-19 patients [6,7,8]. 

CQ and HCQ have a core structure consisting of two aromatic rings fused, the 4-aminoquinoline nucleus, and an amphiphilic weak basic side chain (represented in green and red colors in Figure 1, respectively). The two chemical structures differ only for the hydroxyl group at the end of *N*-ethyl side chain (represented in blue color in Figure 1).

These are water-soluble molecules and, for their chemical nature, can pass the cell membranes; however, the presence of the hydroxyl group in the HCQ makes it more polar and less lipophilic [8]. Moreover, the accumulation of CQ and HCQ in intracellular compartments is due principally to the side chain and both have enantiomers (*R* and *S* isomers). Researches also demonstrated that the *R*-HCQ is present in the blood at higher concentrations than *S*-HCQ. These results suggested the stereoselective processes in the metabolism of the molecule [9].

Studies of structure-activity relationships have demonstrated that the halogen substitutions of different 4-aminoquinolinesderivatives reduced the toxicity, but it also reduced the pharmacological activity. Instead, the therapeutic ratio is decreased by substitution of the alkyl side chain with an aryl chain. Nevertheless, the therapeutic ratio decreases and toxicity increases with an increase of the alkyl side-chain length above 5 carbons [7,10].

Usually, CQ and HCQ are administered as phosphate and sulphate salts, respectively, and both drugs are absorbed in the upper intestinal tract. However, clinically, HCQ is more frequently used than CQ for its lesser toxicity [11]. Both molecules give mild nausea, stomach cramps, gastrointestinal upset, and mild diarrhoea as adverse effects and long-term usage determines loss of retinal function and severe retinopathy. These drugs are used, to date, in malaria patients and in several inflammatory diseases like rheumatoid arthritis, Sjögren syndrome, dermatomyositis, and systemic lupus erythematosus [7,8,10]. 

## 2. Results and Discussions

### 2.1. Study Analysis

**Figure 2:**
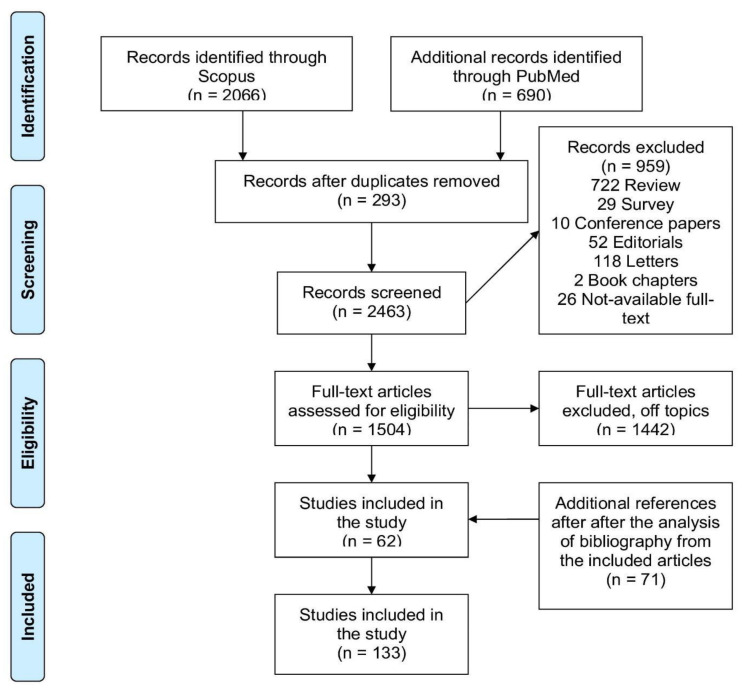
Flowchart detailing literature search according to PRISMA statement.

The initial survey of the literature identified 2756 reports (2066 from Scopus and 690 from PubMed). Then, 293 the duplicates between the two databases were eliminated and were considered only one at a time, resulting in 2463 articles. After the primary screening based on titles and abstracts, 959 manuscripts were excluded since they did not fulfil the inclusion criteria or were off-topic. Finally, 1504 articles were thoroughly analyzed and among these, 1442 were excluded. The analysis of the reference lists from some selected items led to the inclusion of an extra 71 appropriate articles, after titles, abstracts, and full-text study. In total, 133 papers were selected for data extraction. A flowchart illustrating the steps of the study selection is shown in Figure 2. 

**Figure 3:**
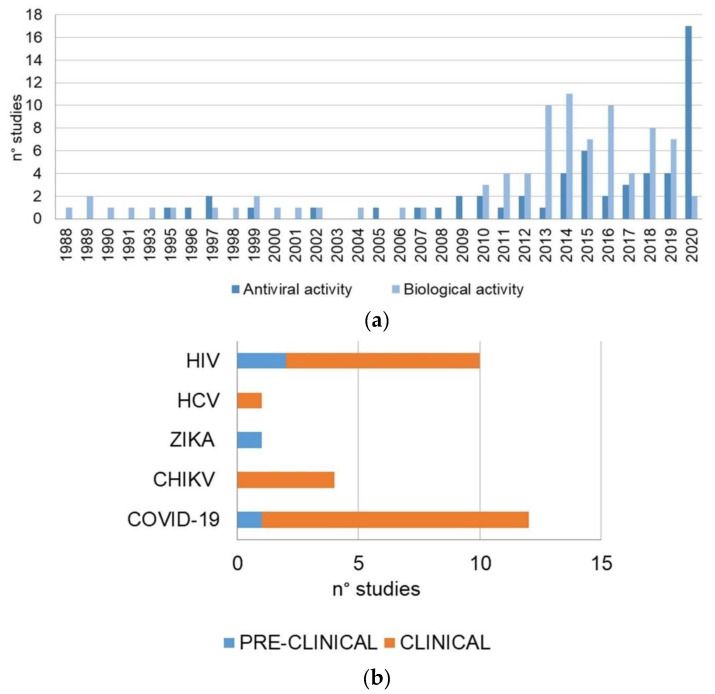
Distribution of the selected studies by year of publication focusing on antiviral activity or other biological activities. (**a**) Distribution, and the total number of preclinical and clinical studies per virus (**b**) HIV = herpes virus simple, HCV = hepatitis C virus, ZIKA = Zika virus, CHIKV = Chikungunya virus, COVID-19 = new Coronavirus Disease 2019; n° = number.

In our review, the articles included were also analyzed in relation to the year of publication and the experimental model used. Regarding the chronology of the publications, most of the articles were published in the last decade. The high peaks correspond to the proportional number of papers on antiviral activity in 2020; this result is consistent with the actual increasing interest towards the role of HCQ as a possible therapeutic strategy in the current COVID-19 sanitary emergency. HCQ alone, or in combination with other drugs, was used in various types of infections. HIV and COVID-19 were the most cited, with 10 and 7 reports each, respectively. Our systematic review included 49 preclinical studies and 84 clinical trials. The considered in vivo studies were carried out using allograft or xenograft models. The most important outcomes of the review are graphically illustrated in Figure 3.

**Figure 4:**
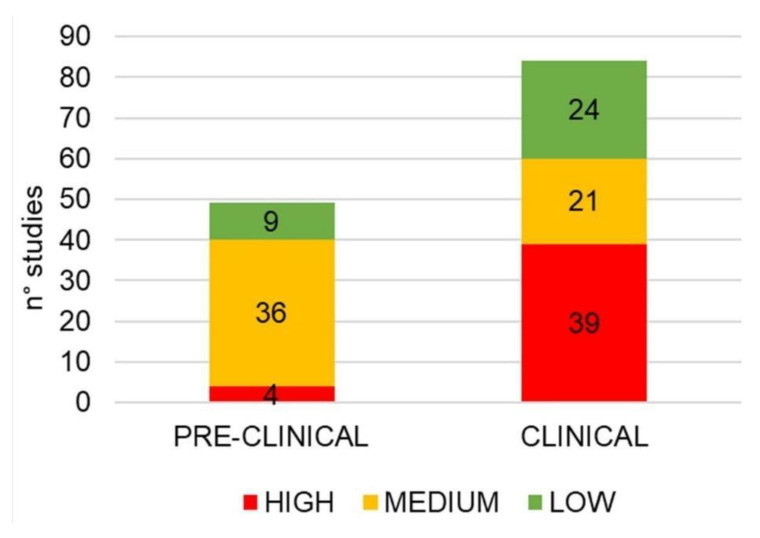
The quality assessment is based on a checklist adapted from the Cochrane Handbook for Systematic Reviews of Interventions. The preclinical and clinical studies have been classified as being of high (red section), medium (yellow section), and low risk (green section) of bias; n° = number.

The assessment of bias risk based on a checklist adapted from the Cochrane Handbook for Systematic Reviews of Interventions is reported in Figure 4. The number of high-risk reports is due to the case reports considered. 

### 2.2. Hydroxychloroquine and Viral Infections

HCQ has been used mainly as an antimalarial drug, but it has also proven effective against viral infections. HCQ demonstrated its antiviral efficacy in inhibiting the endosomal-lysosomal acidification, which is essential for the entry, replication, and infection process of different viruses [12]. In particular, HCQ-induced alkalinization processes cause the expansion and vacuolization of lysosomes, inhibiting their functions. This activity can reduce post-transcriptional protein modification, enzyme release, receptor recycling, activation of cell signaling pathways, and cell membranes repair. As a result, there is the prevention of cell infection.

Further, HCQ antiviral activity is also related to its anti-inflammatory and immunomodulatory effects. Different studies have, indeed, demonstrated that viral diseases are caused by a direct viral infection of susceptible cells and also by an impact on the immune response with consequent uncontrolled release of pro-inflammatory chemokines, cytokines, and other mediators [13]. The most studied antiviral activity of HCQ is that exerted against HIV; however, the current spread of the Coronavirus infection has brought attention back to this drug. 

**Figure 5:**
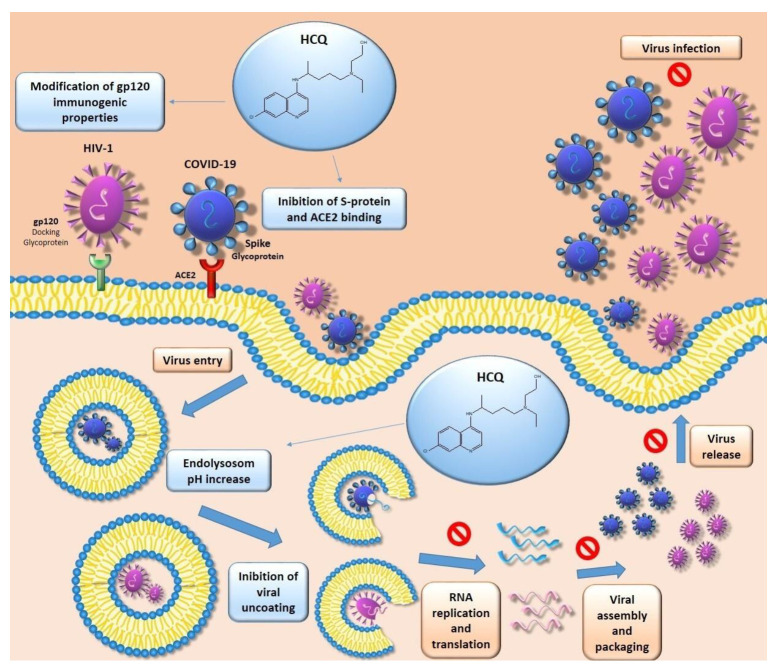
Proposed mechanism for Hydroxychloroquine (HCQ) antiviral activity against COVID-19 and HIV. HCQ seems to block the virus’ entry into the cell by preventing the binding of viruses to the cell surface receptor and increasing the phagolysosome pH, thus interrupting the virus fusion to the host cells. HCQ can also inhibit nucleic acid replication, viral proteins glycosylation, virus assembly, transport of new virus particles, viruses release, and other processes to achieve its antiviral effects [14]. Specifically, the anti-HIV activities are highly linked to the post-translational modification of glycoprotein 120 (gp120). This leads to the loss of gp120 immunogenic properties and reduces new virions infectivity [15,16]. On the other hand, HCQ antiviral activity against COVID-19 seems to be related to its ability to modify the *n*-terminal glycosylation of ACE-2, leading to reduced interaction between ACE-2 and Spike and so to cell infection [8].

In this systematic review, clinical and in vivo studies evaluating HCQ antiviral activity against the Human Immunodeficiency Virus (HIV), Chikungunya Virus (CHIKV), Flavivirus, and Coronavirus have been analyzed (Figure 3B, Figure 5, and Table 1). 

#### 2.2.1. HIV-1

HIV is part of the genus *Lentivirus*, of the Retroviridae family, and it is divided into two lines: HIV-1 and HIV-2. The most virulent and infectious is HIV-1, since it causes most of the HIV infections in the world. The target cells of HIV are those rich in CD4 receptors, such as some lymphocytes called CD4^+^, which play a crucial role in human immunity. Indeed, these lymphocytes activate different immune system cells depending on the type of unwanted host they come in contact with. It seems that acute HIV infection is highly linked to a rapid depletion of CD4^+^ T cells in gut lymphoid tissue. This event is related to an alteration of the intestinal mucosa integrity, resulting in bloodstream translocation of microbial products like lipopolysaccharides (LPS) [42]. It has been hypothesized that LPS, through the binding and activation of Toll-like receptor 4 (TLR-4), is responsible for the immune system activation observed in HIV infection. Although the real HCQ mechanism of action has not been well assessed, it seems that the anti-HIV effects are highly linked to the post-translational modification of glycoprotein 120 (gp120) in monocyte and T cells. Consequently, there was a modification of gp120 immunogenic properties and a reduction of new virions infectivity [15,16]. 

The first randomized, double-blind, placebo-controlled clinical trial about the anti-HIV-1 activity of HCQ was published in 1995. In this study, 40 asymptomatic HIV-infected patients received either 800 mg/day HCQ or placebo for eight weeks. All patients treated with antiretroviral therapy (HAART = zidovudine (ZDV), 2′,3′-dideoxyinosine, or 2′,3′-dideoxycytidine) and stopped taking it four weeks before the clinical trial start. Unlike placebo, at the eighth week, HCQ displayed a reduction in HIV-1 RNA total plasma levels in 12 out of 19 patients. Furthermore, the percentage of CD4^+^ T cells decreased significantly in the placebo group and remained stable during the treatment with HCQ, indicating a probable stabilization of immune function in the HCQ group. HCQ administration also induced a decrease in serum interleukin 6 (IL-6) and immunoglobulin G (IgG). However, no significant changes were found in the IgA and IgM levels [17]. The anti-HIV-1 effect of HCQ was also compared with that of ZDV, a nucleoside reverse transcriptase inhibitor, in a randomized, placebo-controlled clinical trial conducted on 72 HIV-l-infected asymptomatic patients. All subjects were treated for 16 weeks with 800 mg/day HCQ (*n* = 35) or 500 mg/day ZDV (*n* = 37). As in the previous study, patients who had received HAART stopped taking it four weeks before the clinical trial’s outset. After 16 weeks, total plasma HIV-1 RNA levels were reduced in both the ZDV group and HCQ group, although the extent of anti-HIV-l activity in HCQ patients was lower than that observed in ZDV subjects. However, eight subjects in the Azithromycin (AZM) group showed an increase in HIV-1 RNA levels in the 16th week, indicating the rapid emergence of viral resistance. Contrarily, in the HCQ group, increased antiviral activity was revealed after 16 weeks rather than after 8 weeks, and no subject showed an increase in HIV-1 RNA levels at either 8 or 16 weeks of treatment. This evidence suggests that no resistance developed under HCQ therapy or that it might develop more slowly than under ZDV. A reduction in serum p24 antigen levels in both ZDV and HCQ groups was also described, while only in the HCQ group could a decrease in IL-6 and IgG levels be observed [18]. This reduction of IgG levels displayed after HCQ treatment in both studies may be significant since autoantibodies contribute to CD4^+^ cell depletion and autoimmune diseases observed in HIV infection.

Further, as lymphoid tissue is considered the primary site of HIV reservoirs and a critical site affected by CD4^+^ T cells depletion, the HCQ concentration was assessed in the plasma and adenoid tissue (At) of 8 HIV-infected patients administrating 400 or 800 mg of HCQ for eight weeks. After taking these dosages, it was demonstrated that the mean HCQ concentration was significantly higher in At than in plasma [21]. This different drug distribution was also confirmed by an in vivo study using rabbits as an experimental model, receiving 15 mg/kg of HCQ subcutaneously [22]. Thus, the anti-HIV activity of HCQ could be linked to its accumulation in lymphoid tissue, a relevant site for HIV immunopathogenesis and replication.

Since monotherapy is not recommended in treating HIV, HCQ has also been tested in synergy with other drugs commonly used to manage HIV. In this regard, 400 mg/day of HCQ in a combination regimen with 1000 mg/day hydroxyurea and 250–400 mg/day didanosine (dosed per body weight) was administered for 48 weeks to 22 asymptomatic HIV-1 infected patients naïve to antiretroviral treatment. Only 16 out of 22 patients were evaluable. These 16 subjects, at the 12th week, showed a significant reduction in viral load which was maintained until the 48th week. Furthermore, at week 12, an increase in CD4^+^ percentage was also shown, and this improvement was kept until the 48th week [19]. To evaluate the long-term efficacy and safety of HCQ, hydroxyurea, and didanosine combination, they were also tested on 17 HIV-infected naïve patients for 144 weeks. All subjects received 200 mg HCQ, 500 mg hydroxyurea, and 125–200 mg didanosine twice daily. Of the 17 patients who started treatment, 14 remained until the end of the 144th week. After 114 weeks, viral load was reduced by 1.6 Log_10_ copies/mL under baseline (*p* < 0.001), eight patients (47%) had an unnoticeable viral load (< 400 copies/mL), and two patients (12%) had a measurable viral load, but resistance mutations were not detected. Four patients (24%) had both detectable viral load and resistance mutation: one with both 62V and 65R and three with both 74V and 184V mutations; the latter three were assessed as didanosine resistant, while no resistance was found for HCQ. However, in all cases, the viral load remained below the baseline at the 144th week. The CD4^+^ cell count had increased significantly, while the percentage of CD8 cells was reduced up to the 144th week [20]. This HCQ noticeable impact on immune activation, thereby increasing CD4^+^ T cells, was also demonstrated in a prospective study conducted on 20 HIV-infected immunologic no responders treated with standard antiretroviral therapy [23]. These results suggested that the combination of HCQ, hydroxyurea, and didanosine could be a valid alternative to the highly active commercial HAART in HIV-patients. Nonetheless, these latter studies have some limitations, such as the small number of subjects included and the absence of a control group. Therefore, it is not possible to determine the contribution made by HCQ to the overall decrease in viral load obtained from the combination of drugs. Anyway, the potential anti-HIV efficacy of HCQ, when added to existing treatment with an antiretroviral regimen, was also confirmed by a case report about a patient with HLA-B27-associated spondyloarthropathy and HIV infection [43]. 

In contrast to the results mentioned above has been published a randomized, double-blind, placebo-controlled trial performed on 83 patients to which 400 mg HCQ (*n* = 42) or placebo (*n* = 41) were administered for 48 weeks. All patients were naïve to HAART or had stopped this therapy 22 months before the trial began; 17 subjects in the HCQ group and 8 in the placebo group interrupted study medication before the 48th week. At the end of treatment, in the HCQ group, compared to placebo, patients showed a reduction in total CD4 cell count and a significant viral load increased from the 12th week above baseline [24]. Hence, based on these results, HCQ did not decrease immune activation in patients with chronic HIV infection who were naïve to HAART, as there was an increase in HIV viral replication and a negative effect on CD4^+^ cell counts. In light of these results, there was the need to consider that the first two described clinical trials, which reported the antiviral effect of HCQ, were on short-term treatments (8 or 16 weeks) and that they used an HCQ dosage of 800 mg/day [17,18]. In contrast, the latter used only 400 mg/day [24], corresponding to the maximum recommended dose for long-time use. Besides, the latter study also enrolled more patients than the other studies, and unlike the trial of Piconi et al. [23], which described significative effects in reducing immune activation after HCQ administration, was conducted in the absence of antiretroviral treatments [24]. Therefore, further clinical trials involving a larger number of subjects would be necessary to assess the real antiviral activity of HCQ in monotherapy and synergy with antiretrovirals drugs.

Finally, assuming that women resistant to HIV infection show a low activation of the immune system at the level of the female genital tract (FGT), HCQ has been investigated as a drug able to prevent HIV infection. It has been shown that the “immune quiescent” state of HIV-resistant women keeps the immune response against pathogens intact. For this reason, it was thought to induce pharmacologically, in a rabbit model, this immunological quiescence state through an intravaginal implant capable of providing a controlled release of HCQ. Considering that a concentration above 6.48 μg/mL of HCQ was able to interfere with the gp120 glycosylation process, the vaginal implant was projected to release HCQ with the longest possible duration at a concentration greater than 4.34 μg/mL but lower than 21.7 μg/mL. After six days, this implant improved mucosal epithelial integrity, reduced submucosal immune cell recruitment, decreased gene expression and protein of T cell activation markers, and minimized the activation of key pro-inflammatory mediators [25]. Hence, HCQ can be considered a promising drug able to maintain a low baseline level of immune activation and may also play a role in preventing HIV infection. 

#### 2.2.2. Chikungunya Virus

The single-stranded RNA virus, Chikungunya virus (CHIKV), is an alphavirus belonging to the Togaviridae family, spread mainly in America’s regions. The usual CHIKV vectors are rodents, while humans are infected by *Aedes albopictus* and *Aedes aegypti* and mosquito bites. The first incubation stage can vary between 2 and 12 days, and three phases follow it: (1) the acute viraemic phase, characterized by severe polyarthritis, fever, and a rash, generally resolving in three weeks; (2) the post-acute stage, identified by arthritis with the addition of synovial and periarticular inflammation, neuropathy, neuropsychiatric disorders, and peripheral vascular disorders, usually takes its time at the end of three months; (3) the chronic phase that appears when the symptoms of the previous phase do not end after three months. Generally, the acute phase of CHIKV infections is treated with non-steroidal anti-inflammatory drugs (NSAIDs), while for the chronic persistent phase, treatments involving HCQ as monotherapy or combined with methotrexate (MTX) and/or sulfasalazine seem to be effective.

HCQ does not appear to affect the initial stage of infection, as demonstrated in a prospective randomized parallel-group study of 2009. In this trial, combinations of NSAIDs, HCQ, and/or corticosteroids were assessed in patients with classic CHIKV features. A total of 120 subjects were divided into groups treated with 200 mg/day aceclofenac (ACF), 400 mg/day HCQ, and 10 mg/day prednisolone (PRD) in different combinations. Only 114 subjects remained until the end of the trial (six weeks). It was seen that HCQ had no benefit in the early stages of CHIKV infection and also in the reduction of the VAS (used for pain assessment) and in the improvement of Barthel’s indexes (used for instrumental activities of daily living and activities of daily living). In fact, there was no difference between groups treated with ACF + HCQ and ACF alone. Similarly, the combination of ACF + HCQ + PRD did not add any additional benefit over ACF + PRD [26]. 

In contrast, HCQ seems to affect the improvement of CHIKV chronic phase diseases. In a recent multicenter study, the efficacy of HCQ was evaluated in 39 patients with rheumatic manifestation related to CHIKV chronic phase. In four subjects, the treatment was interrupted due to the onset of side effects such as nausea, stomatitis, rash, headache, while the evolution of CHIKV disease was evaluated in only 22 patients. After three months of treatment, evidence of synovitis was disappeared in 10 of 20 subjects (50%) with swollen joins while complete remission was verified in five patients (19.2%) [27]. However, another study demonstrated that the effects of HCQ in combination with MXT and sulfasalazine were superior to those shown in monotherapy. In particular, in a randomized controlled open-label study, the impact of HCQ in monotherapy or association with MTX and sulfasalazine was assessed in 72 patients with chronic persistent chikungunya arthritis. In this trial, 400 mg/day HCQ were administrated to 35 subjects, while a combination of 15 mg/day MTX, 1 g/day sulfasalazine, and 400 mg/day HCQ was administrated to 37 patients. Either treatment lasted 24 weeks and for the first 6 weeks, both groups received an escalated dose of prednisone (7.5 mg/day). At the end of the 24th week, only the combination of drugs improved disease activity and reduced disability and pain [28]. These results, following those obtained in a previous uncontrolled 16-week study, since a reduction in ACR score was shown after MTX (15–20 mg/weekly) and HCQ (400 mg/day) administration to CHIKV infected patients with persistent inflammatory polyarthritis [29].

The results obtained could be explained with the different characterization of virus infection phases. It is known that while the acute phase of CHIKV infections is due to the development of viremia, the chronic phase is closely related to an immune-mediated phenomenon, as pro-inflammatory cytokines and chemokines play important roles in the pathogenesis of chikungunya arthritis [44]. Therefore, considering the results obtained from these clinical studies, it is possible to say that although HCQ does not affect viremia reduction, as demonstrated by the lack of activity in the first phase of infection, it can improve the chronic phase diseases by reducing inflammation.

#### 2.2.3. Flaviviruses

Hepatitis virus, an RNA virus belonging to the Flaviviridae family, is closely related to liver disease, which in 70–80% of patients becomes chronic, resulting in major complications such as cirrhosis and, in the year, also liver cancer. The antiviral activity of HCQ on the hepatitis C virus (HCV) in monotherapy has not been tested. However, it seems that this drug increases the antiviral effect of standard drugs. In a study including 120 Egyptian patients infected by the hepatitis C virus, it was seen that the combination of HCQ with pegylated interferon and ribavirin could improve biochemical and virological responses in chronic hepatitis C subjects. All patients were randomized and divided into two groups; the control group treated with standard-of-care (SOC) consisted of 160 µg of subcutaneous pegylated interferon and 1000–12000 mg/day of oral ribavirin, and the group treated with 200 mg HCQ plus SOC. At the end of the treatment (12 weeks), patients of HCQ + SOC group showed a high virological response compared to the control group (54/60 (90%) *vs*. 43/60 (71.7%); *p* = 0.011). Moreover, in the HCQ + SOC group, there was a normalization of ALT levels, as is also demonstrated by the earlier biochemical response (EBR) highlighted in HCQ + SOC group 58/60 (96.7%) than SOC group 42/60 (70%) [30]. These results were confirmed by several case reports regarding patient treatment with Porphyria Cutanea Tarda and Hepatitis C. It was seen that a low dose of HCQ (100 mg twice weekly) prevented the recurrence of Porphyria Tarda and reduced the viral response during HCV therapy, including ribavirin and interferon [45]. Furthermore, in a patient with chronic hepatitis and rheumatoid arthritis, a low risk for hepatitis virus reactivation was observed after treatment with steroids (< 7.5 mg/day), HCQ or sulfadiazine [46] in combination with antivirals as prophylaxis [47,48].

Another possible antiviral effect of HCQ is exerted on Zika virus (ZIKV), an RNA virus of the Flaviviridae family transmitted by numerous mosquitoes of the genus *Aedes*. No clinical studies have been conducted yet, but an in vivo study has demonstrated the ability of HCQ to attenuate vertical transmission of ZIKV by reducing placental and fetal infection. In this study, pregnant mice were treated with 40 mg/kg/day HCQ via intraperitoneal injection beginning one day after ZIKV infection. It was seen that HCQ acted as an autophagy inhibitor by increasing p62 levels in trophoblast and thus reducing placental ZIKV infection and fetal growth defects [31]. To date, no clinical trials have been conducted to evaluate HCQ antiviral effects in patients infected by ZIKV.

#### 2.2.4. Coronavirus Disease of 2019

Coronaviruses, belonging to the *Coronaviridae* family, are enveloped positive-sense single-stranded RNA viruses highly distributed in humans and vertebrates like bats, which are proposed as their main reservoir. Specifically, Coronavirus Disease of 2019 (COVID-19) was first identified in December 2019 in Wuhan, the capital city of Hubei (China), and it is caused by the SARS-CoV-2 virus [49]. Since COVID-19 has spread rapidly in many countries, it has quickly become a global pandemic, so it is necessary to develop drugs able to exert antiviral activity against it. A recent study showed HCQ in vitro antiviral activity against SARS-CoV-2 (EC_50_ = 0.72 μM) [50]. HCQ seemed to be able to inhibit the first step of the viral replication cycle by interfering with the link between spike (S) viral protein and the angiotensin-converting enzyme 2 (ACE-2) receptor [8]. It would also appear that HCQ was able to induce changes in cell membrane pH resulting in reduced viral entry and inhibition of the last stages of replication. Moreover, HCQ may abolish the cytokine storm related to the advanced stages of COVID-19 through immunomodulatory activity. To date, several clinical studies are underway to evaluate the efficacy of HCQ for the treatment of COVID-19. In a randomized clinical trial conducted in China, 62 patients with COVID-19 were randomly assigned to two groups: the control and the HCQ groups. Either group received standard treatment including antiviral agents, oxygen therapy, immunoglobulin, and antibacterial agents, with or without corticosteroids, but patients in the HCQ group also received oral HCQ 400 mg/day for five days. During treatment, clinical characteristics, clinical recovery time (TTCR), and radiological results were assessed to determine the effect of HCQ. It was seen that in the HCQ group, the recovery time of body temperature was shorter than in the control group and that the cough remission time was also significantly decreased, while only patients in the control group progressed to severe illness. Furthermore, in the HCQ group, 61.3% of patients showed significant absorption of pneumonia [6]. In another open-label non-randomized French clinical trial, the efficiency of HCQ in reducing the viral count was also demonstrated. In this study, 36 subjects were divided into two groups: 16 patients for the control group and 20 subjects for HCQ who were administered 600 mg/day HCQ. Among the HCQ group, six patients also received 500 mg of AZM for the first day, followed by 250 mg/day for the next four days to prevent super bacterial infections. PCR results from nasopharyngeal samples were negative for 70% of patients treated with HCQ and 12.5% in the control group (*p* = 0.001) on day six. Furthermore, when the effect of HCQ as monotherapy was compared to that of HCQ + AZM, it was found that at day six, 100% of HCQ + AZM treated subjects were virologically negative compared with 57.1% of patients treated with HCQ as monotherapy [32]. These results suggest a synergistic effect between HCQ and AZM and are supported by an uncontrolled non-comparative observational study conducted by the same France group, in a cohort of 80 slightly infected patients. In this study, HCQ + AZM treated people were 83% virologically negative on day 7 and 93% on day 8; after 10 days of treatment, only 2 subjects still remain contagious. Furthermore, the average duration of hospitalization was found to be 4.6 days after the administration of HCQ plus AZM [33]. However, these last two studies have a limit, as Gautret et al. carried both out. In particular, in the first study, data are available up to 6 days despite the planned 10 days, and in the second study, 6 patients from the previous study were also included. Gautret also conducted a retrospective analysis of 1061 cases in which, after treatment with HCQ + AZM, good virological care and clinical outcomes were found in 973 patients (91.7%) within 10 days. A prolonged viral carriage was observed in only 47 (4.4%) subjects with a high viral load at the moment of hospitalization, but on day 10 the viral culture was negative. Finally, all but one on day 15 were PCR cleared [51]. By contrast, there are results obtained by several clinical studies that dismiss the possible use of HCQ for the treatment of COVID-19. In particular, a prospective study on 11 severe COVID-19 infected patients treated with the same dosage used by Gautret et al. (600 mg/day HCQ and 500 mg AZM for the first day followed by 250 mg/day from day 2 to 5) was shown to provide no evidence of clinical benefits or a strong antiviral activity through the combination of HCQ and AZM [34]. The ineffectiveness of HCQ has also been declared in a multicenter, open-label, randomized controlled trial involving 150 hospitalized infected patients (148 with mild or moderate disease and 2 with severe disease). All subjects were divided into two groups (in a 1:1 ratio): the control group that received only SOC, consisting of antiviral, glucocorticoid, and antiviral, and the group treated with HCQ plus SOC. The administrated dose of HCQ consisted of 200 mg/day for the first three days, followed by 800 mg/day as maintained dosage until the end of the treatment. After 28 days of treatment, there was no significant difference in SARS-CoV-2 negative conversion in either the HCQ + SOC or the SOC group. Similarly, there were no significant differences in the median time to negative conversion and the probability of symptom alleviation within 28 days between HCQ + SOC and the SOC group [35]. Another multicenter, randomized controlled Egyptian trial evaluated, in 194 subjects with COVID-19, the safety and efficacy of HCQ compared to SOC. In terms of mechanical ventilation need and mortality, the addition of HCQ (400 mg twice daily (in day 1) followed by 200 mg tablets twice daily) to SOC was not associated with an improvement of COVID-19 patients’ health [36]. These results were confirmed by a recent randomized, double-blind, placebo-controlled trial conducted in the USA and Canada. In this study, 419 early and mild COVID-19 subjects were randomly assigned to two groups, the HCQ group treated with 800 mg HCQ once, followed by 600 mg in 6 to 8 h, then 600 mg daily for 4 more days, and the placebo or control group. Within 14 days of treatment, there was no change in the severity of symptoms in non-hospitalized patients between the HCQ group and the placebo group (difference in symptom severity: relative, 12%; absolute, −0.27 points (95% CI, −0.61 to 0.07 points); *p* = 0.117) [37]. Likewise, 2 studies carried out by Mahévas et al. supported the ineffectiveness of HCQ and highlighted that HCQ administration to COVID-10 patients was highly related to ECG modification [38,39]. ECG modification, resulting in QT-interval prolongation, is a characteristic side effect associated with HCQ treatment that has been shown in several clinical studies on patients infected with COVID-19. In particular, the risk of QT-interval prolongation was increased when HCQ was associated with AZM, as demonstrated in a cohort of 84 patients who received 400 mg twice-daily HCQ plus 500 mg/day AZM. In these patients, on day 3.6 ± 1.6 of therapy, the EGC showed a QTc prolongation from a baseline average of 435 ± 24 ms (mean ± SD) to a maximal average value of 463 ± 32 ms. Moreover, in nine subjects (11%) there was a severe prolongation of QTc to > 500 ms (baseline average of 447 ± 30 ms to 527 ± 17 ms (*p* < 0.01 (one-sample *t*-test)) [52]. This complication was also confirmed in a retrospective study of 251 COVID-19 patients treated with HCQ/AZM [53]. It seems that a predictor of extreme QTc prolongation was renal failure and that its incidence increased with longer treatment. The probability of QTc prolongation may also increase in the presence of other factors such as previous cardiovascular diseases, metabolic degeneration (hypoxia, pH, multiorgan system failure, and electrolyte abnormalities), age, and sex (females seem to be more at risk) [54]. Therefore, since QTc prolongation to more than 500 ms is known to be associated with a high risk for malignant arrhythmia and fatal stroke, recent guidance suggests the ECG screening with QTc evaluation in COVID-19-infected patients treated with novel therapies including HCQ/AZM [55]. It has also been suggested that the use of drugs that block late sodium channels (mexiletine or lidocaine) and close attention to serum electrolytes, in addition to the evaluation of heart rate and QT intervals, may allow the administration of HCQ/AZM even in subjects with prolonged QT intervals [54].

Despite the lack of real antiviral evidence related to HCQ administration, this drug has also been investigated as a possible prophylactic agent. In fact, the pharmacokinetics of HCQ, like its long half-life and the high concentration in the lung (500-times higher than blood concentration), has made it an ideal candidate for prophylactic use [56]. The first study conducted on this line was performed in South Korea on 211 virus-exposed individuals, including 189 patients and 22 care-workers. The HCQ administration (400 mg day) as post-exposure prophylaxis resulted in the negative follow-up PCR tests after the end of 14 days of quarantine period (only 97.4% of patients and 95.5% of care-workers completed the study) [40]. However, it is necessary to consider that this is a single-center clinical study with a high risk of bias and that a subsequent randomized clinical study has denied it. In particular, Boulware D.R. et al., in a randomized, double-blind, placebo-controlled trial, tested HCQ as a prophylactic agent within 4 days after virus exposure. There were 821 asymptomatic participants randomly assigned to receive either placebo or HCQ (800 mg once, followed by 600 mg in 6 to 8 h, then 600 mg per day for 4 additional days). After 14 days of treatment, it was demonstrated that HCQ did not prevent COVID-19 infection when compared to placebo, since the incidence of illnesses compatible with Covid-19 disease did not differ significantly between subjects receiving HCQ (49 of 414 (11.8%)) or placebo (58 of 407 (14.3%)). Furthermore, the onset of side effects was more frequent in patients treated with HCQ than placebo (40.1% *vs*. 16.8%) [41]. To better assess the incidence of side effects linked to HCQ administration as post-exposure prophylaxis, a cross-sectional study was conducted among 140 doctors. Sixty nine adverse events were documented in 44 subjects (31%); the most common reported symptoms were headache followed by nausea, dizziness, abdominal cramps, and loose stools, while hypoglycemia was seen in only three diabetic participants [57]. Hence, even if the side effects highlighted were not serious, it is recommended to take the utmost care before using HCQ for COVID-19 chemoprophylaxis. The ineffectiveness of HCQ administration as post-exposure prophylaxis has also been demonstrated by an in vivo study on macaques. Maisonnasse P. et al. tested different treatment strategies, including HCQ alone or in combination with AZM, in comparison to placebo. All the treatments were administrated before or after viral load. It was seen that when HCQ was administrated as pre-exposure prophylaxis, it did not protect against the acquisition of the infection. Similarly, neither HCQ nor HCQ + AZM had beneficial effects in improving viral infection’s symptoms [58], confirming previously analyzed clinical studies’ negative results. Several case reports have supported all these results since people already using HCQ for a long time to treat inflammatory diseases also showed severe illness related to COVID-19 [59,60]. 

In the light of collected data, despite the success of the first clinical trials, the latest studies have shown the ineffectiveness of HCQ for the treatment and prevention of COVID-19 infection. So that, if the U.S. Food and Drug Administration (FDA) had initially authorized the use of HCQ in case of emergency [61], in June 2020, the FDA revoked this authorization [62] since the potential HCQ effectiveness in treating COVID-19 was overtaken by severe cardiac adverse events and other serious side effects. In fact, there is the need to consider that in subjects with severe COVID-19, the abundance of inflammatory molecules like interleukins and tumor necrosis factors generate a cytokine storm, leading to a septic shock and multiple organ failure. In hepatic and renal dysfunction, HCQ metabolism and clearance were compromised and its safety is altered. Moreover, recently the Surviving Sepsis Campaign guidelines on the management of critically ill patients with COVID-19 concluded that there was insufficient evidence to recommend the routine use of HCQ in patients admitted in ICU [63]. In the same way, the American College of Physicians practice guidelines do not recommend HCQ for prophylaxis or treatment [64]. International trials like SOLIDARITY (International trial by World Health Organisation) [65], RECOVERY (Randomised Evaluation of Covid-19 Therapy) [66], and DISCOVERY (Trial of Treatments for COVID-19 in Hospitalized Adults) [67] have also stopped the HCQ arm. In particular, the World Health Organisation (WHO), in the SOLIDARITY trial project, has arrested all arms involving HCQ, as evidence showed that it did not reduce the mortality of hospitalized COVID-19 patients compared with SOC. However, this decision was not applied to HCQ use or evaluation in pre- or post-exposure prophylaxis for subjects exposed to COVID-19 [68]. 

To conclude, the HCQ treatment of SARS-CoV-2 infection was not met with its hoped success. This is probably related to the inability of the dosing regimens currently in use to achieve the blood concentration required for the HCQ antiviral activity. Initially, based on physiological pharmacokinetic models, Yao et al. recommended for SARS-CoV-2 infection a loading oral HCQ dose of 400 mg twice daily, followed by a maintenance dose of 200 mg administered twice daily for four days [50]. However, this recommended HCQ dosage regimen was based only on the ratio of free lung trough concentration to in vitro EC_50_ values (the EC_50_ of HCQ for SARS-CoV-2 ranged between 0.72 and 17.31µM) and did not consider the tendency of HCQ to accumulate within acidic cellular organelles like endosomes, lysosomes, and Golgi apparatus [69]. In fact, it has been demonstrated that HCQ concentration in lysosomes is higher than the extracellular concentration (80 µM *vs*. 0.5 µM, respectively) [70]. Based on these results, it was considered necessary to compare the EC_50_ values obtained in vitro with the plasma concentration and not with the lung concentration. In a study investigating HCQ pharmacokinetics in COVID-19 patients treated with 600 mg/day of HCQ, it was found that the mean blood concentration of HCQ was 0.46 mg/day [32], which was below the lowest estimated levels of 0.48 mg/mL corresponding to the in vitro concentration of 0.72 µM. Further, a plasma concentration predicted for HCQ antiviral EC_50_ made by Garcia-Cremades et al. found that it should be 4,7 µM corresponding to 1.58 mg/mL, which is much higher than in vivo plasmatic values. To reach this plasma concentration, it should be necessary to take an amount of HCQ higher than 400 mg twice a day for five days or more [71], which would increase the onset of side effects. Thus, the ineffectiveness of HCQ antiviral activity again SARS-CoV-2 can be related to the low current dosing regimens and the impossibility to increase the administered doses due to the increased risk of severe side effects, especially QT prolongation.

### 2.3. Hydroxychloroquine Biological Activity

**Figure 6:**
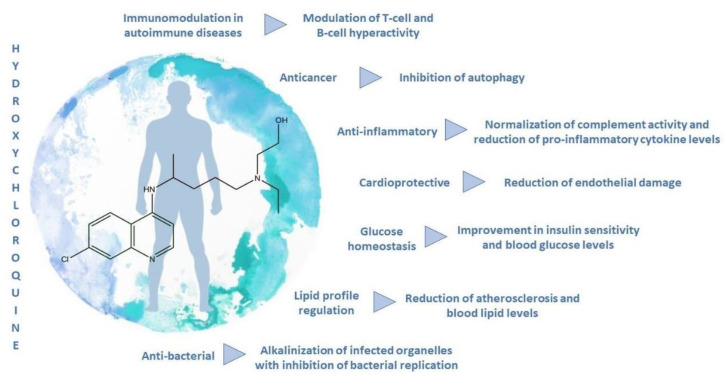
Major biological activities of Hydroxychloroquine (HCQ) and its relative mechanisms of action.

Besides the antiviral effects, HCQ possesses several other demonstrated biological activities (Figure 6). 

**Figure 7:**
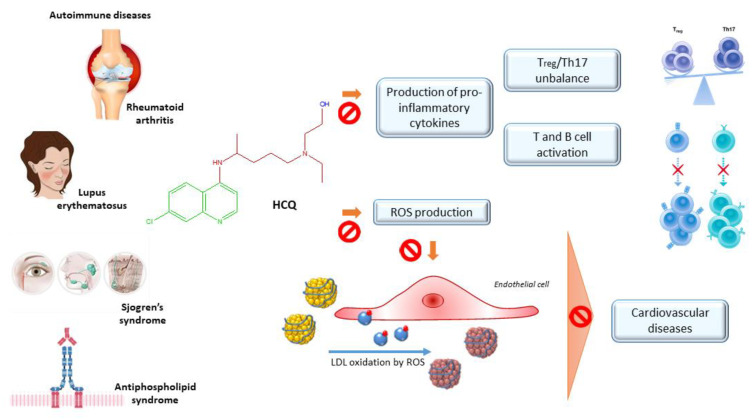
Effects of Hydroxychloroquine (HCQ) on immune diseases and cardiovascular-associated complications.

Most of all, it showed immunosuppressive properties, allowing its employment (alone or in combination) in the first-line treatment of several auto-immune diseases, like rheumatoid arthritis, lupus erythematosus, primary Sjögren’s syndrome, and anti-phospholipid syndrome. Moreover, HCQ was revealed to be effective in preclinical and clinical trials to dampening autoimmune disease-dependent cardiovascular complications (Figure 7), as well as in the amelioration of disease-independent hyperglycemia, hyperlipidemia, and gastrointestinal complaints. 

HCQ exerts anticancer effects by acting synergistically with common chemotherapic drugs. Although it has a well-defined and favorable toxicity profile, the necessity of increasing the dose, in some cases, limits the utilization, due to the toxicity, mainly at cardiac and ocular levels. A two-compartment model, with first-order absorption and a lag time, describes the pharmacokinetics of HCQ. The long-terminal half-life prolongs the time to reach steady-state concentrations, then delays the therapeutic effects. The next-generation formulations allow modulating the pharmacokinetics of HCQ. Avoiding systemic absorption, then liver first-pass metabolism, HCQ may be used in site-specific inflammation, without toxicity [72,73,74]. The selected studies’ primary outcomes are the extent to which HCQ may limit disease progression or exacerbations (Table 2).

#### 2.3.1. Rheumatoid Arthritis

Rheumatoid arthritis is a systemic autoimmune disease, characterized by chronic inflammation and damage to the joints. Immune dysregulation underlies the pathogenesis of rheumatoid arthritis, leading to uncontrolled production of antibodies, mainly rheumatoid factor and citrullinate, involved in the autoreactivity against cartilage and bone [117]. It is estimated that the prevalence of rheumatoid arthritis is around 1%, mainly in women [118]. The immunosuppressive effects of HCQ are due to the ability to modulate T-cell and B-cell hyperactivity, resulting in a reduction of pro-inflammatory cytokine gene expression. As the involvement of neutrophils in this disease, Jancinova, Pazourekova, Lucova, Perecko, Mihalova, Bauerova, Nosal and Drabikova [75] investigated the impact of oral HCQ administration on these cells, in rats with adjuvant arthritis. At doses of 40 mg/kg daily, per oral administration (p.o.), it strongly decreased the blood concentration of neutrophil-derived oxidants, involved in the tissue damage and the onset of chronic inflammation [75].

A 36-week randomized, double-blind, placebo-controlled trial has evaluated the effect of the administration of HCQ in a dose of up to 7 mg/kg per day (maximum 400 mg/day) in patients with rheumatoid arthritis. Within 36 weeks and during the study, HCQ showed statistically significant benefits on physical function, mainly on synovitis and joint pain, without side effects with respect to the control. Moreover, HCQ was associated with a decrement of corticosteroid injections. By contrast, no improvements in psychological function have been demonstrated [76]. Two comparative double-blind, randomized trials, involving 60 patients each, with sulfasalazine and HCQ have demonstrated that HCQ showed no significant differences among overall clinical effects respect to sulfasalazine, but presented a later onset. In the first study, patients treated with HCQ (400 mg/day for 6 months and 200 mg/day for the next 6 months) experienced time-dependent statistically significant improvements in morning stiffness, pain, swollen joints, together with a decrease in blood levels of immunoglobulin M and erythrocyte sedimentation rate [77], while, in the second study, the same treatment was associated to no erosions in the 12% of the patients [78].

In patients with rheumatoid arthritis, the cardiovascular risk is more than doubled, compared to the healthy population. Chronic inflammatory status leads to an intensification of the atherosclerotic process, resulting in a higher susceptibility to hypertension, obesity, and metabolic syndrome. The overproduction of IL-6 is strictly related to lipid profile alterations, given its role in adipose tissue lipolysis. The inflammation and endothelial damage are exacerbated by leptin production. Batun-Garrido, Salas-Magana and Juarez-Rojop [79] found a significant correlation between HCQ treatment and lower IL-6 and leptin levels. The positive effect of HCQ on dyslipidemia was also confirmed by Morris, Wasko, Antohe, Sartorius, Kirchner, Dancea and Bili [80] in a cohort study involving 706 rheumatoid arthritis diagnosed patients, finding a significant and stable cholesterol-lowering, mainly Low-Density Lipoprotein (LDL), and triglyceride decrease associated with HCQ intake (6.5 mg/kg/day). A small but statistically significant amelioration in total cholesterol and LDL under HCQ treatment at the same dosage was also highlighted by a randomized, double-blind cross-over trial on patients with rheumatoid arthritis [81]. The correlation between HCQ and cardiovascular risk was also assessed in a cross-sectional observational study involving 177 women with rheumatoid arthritis. At doses of 200 mg/kg/day, HCQ usage led to lower fasting glucose in women, arising as a valuable tool to enhance glycemic control [82]. However, apparent different outcomes were derived from a randomized double-blind crossover trial, recruiting 23 non-diabetic subjects with stable rheumatoid arthritis to receive 6.5 mg/kg/day of HCQ for eight weeks. For these patients, no significant changes in insulin resistance were observable [81]. This study evaluated only insulin sensitivity, by Homeostatic Model Assessment (HOMA) index, without considering insulin metabolism. Thus, inconsistency should be explained by this factor, together with the short duration of treatment. The anti-diabetic properties of HCQ have been also assessed in patients without arthritis, as reported in the next paragraphs.

#### 2.3.2. Lupus Erythematosus

Systemic lupus erythematosus is a multisystemic autoimmune chronic inflammatory disorder that mainly involves joints, mucosae, skin, and endothelial vessels [90]. For a long time, HCQ has played a marginal role in the overall management of the disease. Since the 90s, the first evidence of HCQ effectiveness in controlling lupus erythematosus manifestations allowed its employment as a first-line medicament. Although it was not recommended in single therapy, the immunomodulating properties of HCQ seem to play an important role in the disease pathogenesis. It is associated with a decrement of exacerbation events, as well as protective effects towards vascular and thrombotic events [85]. Indeed, a 24-week randomized, double-blind placebo-controlled trial demonstrated that the discontinuing of HCQ treatment (100–400 mg/kg/day for at least six months) increased the risk of exacerbations by 2.5 times in patients with quiescent lupus. People who interrupted the therapy exhibited constitutional symptoms of the disease, as well as skin rashes, arthritis, and ulcers [83]. A long-term randomized study evaluated the withdrawal effects of HCQ on quiescent lupus erythematosus patients, revealing that a reduction by up to 57% is associated with HCQ maintaining treatment (272 mg/day) [84]. A case-control study was carried out in order to define the role of HCQ in the survival of individuals affected by lupus. The positive correlation between HCQ and survival led to the consideration of this drug as a great therapeutic option at the proper dose (6.5 mg/kg/bw) in lupus management [85]. If these clinical trials demonstrated the advantages of keeping up the therapy with HCQ in preventing disease exacerbations, doubts persisted about the efficacy of this treatment in the control of more severe clinical forms [83]. As the important role of the imbalance between immune cell populations, several preclinical and clinical studies have evaluated the role of HCQ in restoring this equilibrium. In particular, elevated levels of effector lymphocyte T (Th17) that mediate the autoimmune answer and decreased levels of regulatory lymphocyte T (Treg) that guarantees the immune homeostasis, may be observed in autoimmune diseases, in particular in lupus. Lately, autophagy had risen among the emerging strategies to reestablish the immune balance. As HCQ is a well-known autophagy inhibitor, An, N. et al. [86] evaluated the influence of HCQ intake (100 mg/kg/day) in MRL*/lpr* mice with the lupus-like disease. After four weeks of treatment, HCQ clearly restored the immune balance, by both inhibiting Th17 response and enhancing Treg immunosuppressive effects. The levels of autoantibodies and the expression of inflammatory cytokines, mainly in Th17 cells, were remarkably lowered, due to the inhibition of the activated autophagy, as demonstrated by the increase of autophagic flux marker expression in Th17 and Treg, compared with controls. This randomized trial further evidenced that HCQ treatment also remarkably attenuated kidney inflammation, by limiting the migration of lymphoplasmacytic cells into renal tissues [86]. Preclinical outcomes have been confirmed by a prospective cohort study, involving 41 patients with a diagnosis of lupus treated with 400 mg/day of HCQ. Dysregulated cytokine and autoantibody production, deriving from high autoreactivity that characterizes lupus disease, has been restored by two months of HCQ administration, demonstrating its ability in modulating inflammatory response, with normalized complement activity and reduced levels of pro-inflammatory cytokines, mainly IL-6 and TNF-α [87]. In a multiethnic US cohort on 35 lupus patients, HCQ treatment resulted in significant clinical benefits towards disease progression, probably due to the inhibition of toll-like receptor activation, resulting in down-regulation of IFN-α, which plays a pivotal role in lupus pathogenesis [88].

Infiltrating cells, most of all mast cells, could involve skin tissues, causing one of the most common signs of lupus, skin rashes. The consequences of HCQ intake on skin lesions have been investigated on a MRL/*lpr* murine model of lupus at low (4 mg/kg/day) and high (40 mg/kg/day) oral doses. The number of mast cells decreased with respect to the drinking-water control (from 81 to 50 in low-dose and 12 in high dose), while the mortality rate decreased by up to three times in both treated groups with respect to the control. These in vivo results, together with a significant histopathological alteration regression, suggest that HCQ is a good tool against skin injury in lupus erythematosus [89].

The chronic inflammatory status that features lupus erythematosus leads to a higher susceptibility to cardiovascular complications. Lupus, indeed, is often characterized by endothelial dysfunction, the earliest marker of cardiovascular disease, as well as hypertension and renal damage. In a NZB/W F1 mice model of lupus, oral HCQ gavage of 10 mg/kg/day for five weeks reduced the incidence of thromboembolic events. Moreover, improvements in hypertension, renal damage, and heart hypertrophy occurred, probably due to the normalized endothelium response and reduction of Reactive Oxygen Species (ROS) attributable to HCQ intake [90]. The same effect of normalizing nitric oxide and ROS production have been confirmed in a NZB/W F1 murine model at different times. HCQ at 3 mg/kg/day p.o. protected vascular endothelium, with a strong improvement of endothelial dysfunction [91]. The benefits on atherosclerosis also pass through the lipid-lowering power of HCQ. A clinical study on 155 autoimmune patients revealed a statistically significant association between HCQ (400 mg/day) and a lessening of triglyceride and cholesterol levels, mainly LDL, while no HDL changes were observed [92]. By contrast, HCQ in patients with a mild or inactive condition had no significant effects on lipid profile. A survey involving 65 Chinese lupus patients, treated with HCQ (244 ± 86 mg/day), demonstrated that only triglycerides tended to be lowered, while no statistically significant changes are observable in cholesterol levels [119]. The use of HCQ may be helpful in minimizing cardiovascular risk by improving glycemic homeostasis in lupus patients. A cross-sectional study performed between 2000 and 2005 on 149 nondiabetic women affected by lupus estimated that a mean dose of 400 mg of HCQ affects insulin sensitivity and resistance, as assessed by HOMA index, as well as fasting glucose levels [82].

HCQ remains a worthwhile primary or additional therapy in lupus patients, considering the low cost and its safety profile, also in pregnancy. A randomized double-blind study reported the safety of HCQ during pregnancy, correlating this drug with less disease activity and a lower required dose of prednisone [93]. A 5-year prospective study evaluated the effect of HCQ discontinuation on lupus progression in pregnant women. As it occurs in no pregnant people, interruption of HCQ treatment is linked to an exacerbation of the disease. Moreover, there are no statistically significant differences regarding pregnant complications with respect to the control, showing no fetal toxicity at a dose of 6.5 mg/kg/day in breast milk [94]. Fetal safety has been also assessed in women with lupus nephritis by a multicenter study, reporting a reduction of 85% of the possibility of having a small for gestational age baby in patients under HCQ. Moreover, it exerted protective effects on fetal growth [120].

#### 2.3.3. Antiphospholipid Syndrome

Antiphospholipid syndrome is an autoimmune disorder characterized by antiphospholipid autoantibodies production. If it is not associated with other autoimmune diseases, it is called primary [97]. The incidence of the pathology is greater in young women of reproductive age and it often has a negative impact on pregnancy, with unfortunate outcomes due to the development of placental ischemic pathologies. Resonance spectroscopy, indeed, revealed that the fetal brain and placenta are the main targets of autoantibodies localization [95]. As complement activation plays a central role in the occurrence of the disease, many studies have evaluated the role of HCQ in inhibiting complement activation. In a mouse model of obstetric antiphospholipid syndrome, HCQ, administered in a daily dose of 200 μg per mouse limited placental abnormalities, with an increase of fetal survival, by inhibiting complement activation [95]. A case report on the use of HCQ on a pregnant woman with recurrent venous thromboembolism confirmed the efficacy of HCQ also in clinical practice. The addition of 400 mg daily of HCQ to a common therapeutic regimen of aspirin and heparin dramatically reduced the episodes of vascular thrombosis [96], showing great antithrombotic properties. Given these results, the mechanisms underlying the use of HCQ in thromboprophylaxis were assessed. Two similar preclinical studies, using one-week treatment with 12 μg/g/day of HCQ and three-weeks treatment with 20 mg/kg/day of HCQ, respectively, were in accordance to assess that the overall amelioration of thrombotic status in mice models of antiphospholipid syndrome was linked to endothelial function improvement by modulating the expression of nitric oxide synthase [97,98]. Moreover, the efficacy of HCQ in antithrombotic therapy may lie in the interference in the coagulation cascade. HCQ, indeed, was revealed to decrease the levels of soluble tissue factor, a key initiator of the process, in patients with antiphospholipid syndrome after three months of therapy with a daily dose of HCQ of 200 mg [99].

#### 2.3.4. Sjögren Syndrome

Sjögren syndrome is an autoimmune disease with a strong negative impact on the quality of life of affected people. The main features of the disease are lymphocytic inflammation and alterations in major salivary glands, causing xerostomia [103]. Even if preliminary results about HCQ use in Sjögren syndrome were not encouraging, to date it arises as one of the first-line drugs in disease treatment. Indeed, an earlier prospective, a two-year double-blind crossover trial on 19 subjects correlated an annual intake of a dose of 400 mg/day with no significant improvements in clinical symptoms and signs of pathology, including tear and salivary gland activity, respect to the placebo [121]. However, a few years later, the first evidence of HCQ effectiveness in Sjögren syndrome treatment was reported. Annual treatment with a dose of 200 mg/day of HCQ showed anti-lymphoproliferative and anti-inflammatory effects, with a reduction of IgG and IgA immunoglobulins, anti-Sjögren autoantibodies, and erythrocyte sedimentation rates [100]. Moreover, the salivary flow rate increased in Sjögren syndrome women who received a daily dose of 400 mg of HCQ for 30 weeks [101], while eye dryness was alleviated by HCQ administration (6.5 mg/kg), as demonstrated by a prospective study on 32 patients [102]. Hypo-salivation deriving from acinar atrophy and fibrosis of salivary glands is often associated with over-expression of TGF-β (transforming growth factor-β). Treatment with HCQ downregulated TGF-β levels in a randomized trial on NOD mice exposed to doses of 50 mg/kg/day intragastrically (i.g.) for 16 weeks, with significant results in delaying loss of saliva secretory function. Moreover, HCQ intake was also accompanied by a decrease in autoantibody production and a lower lymphocytic infiltration [103]. These findings were confirmed by Wu, Pu, Yu and Li [104], showing that 8-weeks treatment with HCQ administered at a dose of 60 mg/kg i.g. in 40 randomized NOD mice led to lower lymphocytic infiltration, with a significant improvement in pathological changes in submandibular gland morphology.

#### 2.3.5. Diabetes

As has already been demonstrated for autoimmune patients, HCQ demonstrated great anti-diabetic properties. The first proofs of the role of HCQ in glucose and insulin homeostasis date back to 1999, when Emami, Gerstein, Pasutto, and Jamali [105] demonstrated that diabetic rats treated with oral doses of 80, 120, and 160 mg/kg/day of HCQ exhibited a dose-dependent increase in insulin blood levels, with a consequent reduction of glucose concentration. Higher doses of HCQ (200 mg/kg/day) were tested by Abdel-Hamid, A.A. and El-Firgany Ael, D. [106] on diabetic rats, finding an HCQ-mediated decrease in the pancreas, as the mechanism underlying the improvement of the metabolic profile in diabetic rats. The same authors associated the beneficial impact of HCQ on insulin resistance in diabetic rats with its ability to restore adipokine balance and reduce endothelial stress markers [113]. Given the positive outcomes deriving from preclinical studies, the therapeutic potential of HCQ was also assessed in several clinical trials. Included in a randomized, double-blinded study of 18 months with 300 mg of HCQ twice a day were 135 diabetic obese patients. HCQ treatment improved glycemic control, as demonstrated by the decrease of glycated hemoglobin by up to 1% respect to the placebo, without any side effects [107]. An open-label longitudinal study engaging 13 obese non-diabetic individuals examined the effects of a dose of 6.5 mg/kg/day of HCQ for six weeks, demonstrating a significant reduction in insulin resistance, assessed by HOMA index [108]. In a randomized, double-blinded, controlled trial on 39 prediabetic subjects, the effect of 12-week treatment with 6.5 mg/kg/day of HCQ on glycemic status and lipidic profile was evaluated. Results reported a significant increase in insulin levels, demonstrating the potential use of HCQ to counteract the risk of developing diabetes [109]. A randomized double-blind study involving 267 type-2 diabetic patients compared the efficacy of HCQ (400 mg/day) and pioglitazone, a common anti-diabetic drug, in the control of glycemic and lipidic profiles. No statistically significant differences emerged between the two medicines in terms of glycated hemoglobin and glucose levels, although both drugs produced an improvement in glycemic parameters. Regarding lipidic status, total cholesterol and LDL levels were reduced more by HCQ than pioglitazone. Given the good tolerability of the treatment, HCQ may arise as a therapeutic alternative in diabetes management [110].

#### 2.3.6. Others (Cancer, Inflammation, Cardiovascular Diseases)

Given the well-recognized properties of HCQ against inflammation, it is easily intuitable that this agent could possess interesting insights into cancer treatment. Chronic intestinal inflammation predisposes to the risk of colitis-associated colorectal cancer. In vivo, HCQ was demonstrated to interfere with cancer growth at different stages of development, both preventing tumorigenesis in the early phases and inhibiting tumor growth in the late phases in mice treated with azoxymethane and dextran sodium sulfate to induce cancer. In terms of animal survival, 120 days treatment with 50 mg/kg of HCQ intraperitoneal injection (i.p.) almost restored the survival rate to pre-treatment values and reduced the size of the tumor. The therapeutic effects of HCQ may be attributed to the significant inhibition of pro-tumorigenic and pro-inflammatory cytokines, which not only limited the tumor progression by reducing inflammation of lamina propria, but also decreased the ROS production in macrophages [111]. Many others are the mechanisms by which HCQ exerts anticancer effects, mainly in synergism with conventional chemotherapic drugs, as discussed later.

Regarding the cardioprotective effect, this review has already focused on the positive impact of HCQ on cardiovascular issues in autoimmune patients. A protective effect of HCQ on neonatal rat cardiomyocytes was proven by Bourke, McCormick, Taylor, Pericleous, Blanchet, Costedoat-Chalumeau, Stuckey, Lythgoe, Stephanou and Ioannou [112] in ischemia-reperfusion animal models. The pharmacological preconditioning with HCQ seems to be a good strategy to protect from ischemia-reperfusion injury. The pretreatment with daily gavage of 200 mg/kg of HCQ, indeed, reduced the cardiac infarct size by 47%. The mechanism underlying this effect is linked to the inhibition of apoptosis and total cell death in neonatal rat cardiomyocytes. The atherosclerotic process contributes to increasing the risk of heart failure. The etiology of this condition is still not clear. A hypothesis supposes that the accumulation of lipids in vessels caused the formation of atherosclerotic plaques that are responsible for vessel narrowing, shear stress, and platelet aggregation. HCQ decreased free-fatty acids, triglycerides, total cholesterol, and LDL levels in diabetic rats under doses of 200 mg/kg/day [106]. Moreover, HCQ (10 mg/kg/day) was demonstrated to exhibit functional and structural protection in 40 high-fat diet mice, by reducing atherosclerotic area by 60% with respect to the control [114]. These favorable effects at the metabolic level might be due to its anti-inflammatory power that influences many other biological activities. In gastrointestinal inflammations, mainly in inflammatory bowel disease, HCQ suppressed pro-inflammatory cytokines and enhanced the expression of ILs involved in anti-inflammatory processes. In mouse models of colitis, the HCQ methacryloylated form (30 mg/kg) avoided systemic absorption, accumulating in the gastrointestinal tract, where alterations in the immune homeostasis of the intestinal mucosa had a positive impact on the disease [74]. Inflammation, together with alterations in the immune system, are at the basis of pulmonary hypertension. The ability of HCQ to interfere with the production of pro-inflammatory cytokines from monocytes and lymphocytes might underlie the observed improvements in systolic pressure and ventricular hypertrophy, in rats with pulmonary hypertension treated with 50 mg/kg/day i.p. of HCQ for 20 days [115]. Likewise, in endometriosis, the abnormal presence of endometrium in other organs leads to a chronic inflammatory status that could be affected by HCQ intervention. Ruiz, Rockfield, Taran, Haller, Engelman, Flores, Panina-Bordignon and Nanjundan [116] observed an increment of peritoneal macrophages in mouse models of endometriosis under HCQ (60 mg/kg i.p.). In their role of scavengers, abnormalities in these cell populations may lead to an accumulation of endometrial cells, with impairment of the disease. Moreover, histopathologic improvement of lesions was observed, probably due to the inhibition of autophagy by HCQ that alters anoikis response of endometrial cells [116].

### 2.4. Hydroxychloroquine and Synergic Effect

The choice of HCQ as an additive therapy in many medical regimens is due to the synergistic effect that enhances the efficacy of other drugs in the treatment of several diseases that have been frequently demonstrated as follows.

#### 2.4.1. Autoimmune Diseases

HCQ belongs to the group of the disease-modifying antirheumatic drug (DMARD), which comprises drugs that are not chemically relted, sharing the same efficacy in dampening the progression of rheumatoid arthritis [122]. It often occurs also that glucocorticoids or natural antioxidant substances are included in the coadjutant therapy of rheumatoid arthritis [123]. A multicenter, randomized clinical trial analyzed the tolerability and the efficacy of combined therapy, including HCQ, sulphasalazine, MXT, and PRD with respect to the use of a single antirheumatic drug in the caring of early rheumatoid arthritis for two years. A total of 195 patients were equally divided into two groups to follow the assigned therapeutic protocol. The primary aim of clinical remission was achieved after one year by 24 of the 97 patients under combinatory therapy, while only by 11 of 98 single-drug therapy users, but this trend was further confirmed during the second year. After one year, 75% of subjects under combinatory therapy and 60% of those under single-therapy reached clinical improvement, intended as 50% clinical response. In terms of tolerability, the cotreatment resulted not to be more dangerous with respect to the single drug [123]. In a prospective trial, patients with the diagnosis of rheumatoid arthritis were scheduled to receive cotreatment of sulphasalazine (1–3 g/day), MXT (7.5–15 mg/week), and HCQ (200 mg/day) for six months. Significant improvements in clinical parameters revealed the efficacy of the cotreatment in counteracting endothelial injury. Indeed, the blood concentrations of endothelial injury markers, mainly soluble E selectin and thrombomodulin, experienced a significant drop after the cotreatment [124]. Likewise, a single-blinded clinical trial on 281 patients confirmed the better therapeutic efficiency of cotreatment (25 mg/week MXT, 2 g/day of sulphasalazine, and 400 mg/day HCQ p.o. plus intramuscular injection (i.m.) doses of 120 mg of methylprednisolone or 80 mg of triamcinolone) with respect to single therapy after three months, without significant differences in side effects [125]. Proofs of the better antirheumatic potential of the combination of drugs with respect to single therapy derived from an observational study that evaluated the higher improvement of quality of patients’ life after one year of coadministration of MXT, HCQ, and corticosteroids with respect to single MXT, or HCQ, or their combination with corticosteroids [126]. Great insights in disease remission were provided by a clinical trial involving 17 patients with active rheumatoid arthritis where the chronic inflammatory status of joints was evaluated through the ^18^F-FDG PET method. It was found that cotreatment with 7.5–15 mg/week of MXT, 2 g/day of sulphasalazine, 5 mg/kg/day of HCQ and a low dose of oral PRD (under 10 mg/day) is associated with a reduction of 30% in ^18^F-FDG uptake measures on PET imaging in 81% of patients after four weeks [127]. Although HCQ is effective and well-tolerated, other therapeutic alternatives have emerged in recent years. Among them, monoclonal antibodies are the most promising. In a multicenter open-label clinical trial, performed on 60 patients with juvenile idiopathic arthritis for 54 weeks, the combination of infliximab monoclonal antibodies with a conventional antirheumatic drug provided the better response in terms of disease inactivation with respect to DMARD administration [128].

In addition to rheumatoid arthritis patients, HCQ is also used in lupus erythematosus, another autoimmune disease. Regarding lupus erythematosus, HCQ is widely used. In vivo studies on a NZB/W F1 murine model of lupus showed that HCQ in combination with PRD (2.5 mg/kg/day and 1 mg/kg/day p.o.) decreased autoantibody production, as well as being able to inhibit toll-like receptor activation, resulting in the down-regulation of type I interferon (IFN-α) which is deeply implicated in lupus pathogenesis. The efficacy of treatment is due to the ability of drugs to alter the expression of urinary and immune cell micro RNA that contribute to lupus progression by post-transcriptional modulation of genes involved in the immune response, pro-inflammatory cytokines production and toll-like receptor pathways [129]. In association with low-dose aspirin, HCQ is also indicated for thromboprophylaxis in patients with lupus. The occurrence of thrombotic events was recorded for 13 years in 189 patients, showing that the cardiovascular complications were more frequent in patients treated only with aspirin, while HCQ (up to 600 mg) was associated with a stronger thrombo-protective effect in patients with lupus [130].

Thanks to its immunomodulatory properties, HCQ (20 mg/kg) was revealed to be useful in graft-versus-host disease, in combination with cyclosporine A. They synergistically suppressed T cell response, allowing the reduction of the dose of drugs in mice [131].

#### 2.4.2. Cardiovascular Risk Management

HCQ revealed the great potential in the management of cardiovascular risk by controlling glucose homeostasis and lipidic profile. Until now, in this review, we discussed the effect of HCQ alone to counteract cardiovascular complications, mostly in autoimmune patients. Here, we reviewed the multiple pleiotropic actions of HCQ in combination with conventional medication in the most common cardiovascular diseases. The cardioprotective effects of one week of treatment with 50 mg/kg of HCQ i.g. against ischemia-reperfusion injury in type-2 diabetic mice were assessed in combination with the phosphodiesterase-5 inhibitor, tadalafil. The synergistic effect reduced the myocardial infarct size by up to 20% and improved insulin secretion and sensitivity [132]. Moreover, low-dose HCQ (3.4 mg/kg/day) prevented cardiomyocyte apoptosis in the periinfarct myocardium, dampening ischemic cardiomyopathy, and cardiac stroke, as demonstrated by Jalal, et al. [133] in rat models. The role of HCQ in the inhibition of platelet aggregation was evaluated in healthy volunteers in comparison with aspirin or clopidogrel. The addition of 400 mg/day of HCQ to aspirin resulted in a significant increase in aggregation inhibition (31%). This inhibition was passed by reducing fibrinogen and inflammatory status by interfering with the arachidonic acid cascade [134].

#### 2.4.3. Anticancer

HCQ explains its antitumor activity thanks to its ability to inhibit autophagy. HCQ is, indeed, an FDA-approved drug inhibiting autophagy [135]. Several types of tumors develop chemoresistance by enhancing autophagic flux. Autophagy consists in the sequestration of materials in autophagic vesicles to be eliminated through lysosomal fusion and allows cells to overcome metabolic and therapeutic stresses. By recycling intracellular components, cells may maintain an energy balance and increase their growth. If it occurs in cancer cells, resistance mechanisms may establish. One of the mechanisms responsible for drug resistance is related to increased drug efflux by ATP-binding cassette (ABC) transporters [136,137]. It has been observed that HCQ is significantly reduced the increase in P-gp (ABCB1) expression and, in combination with several anticancer drugs, induced higher cytotoxicity in refractory cancers by inhibiting autophagic activity [138]. However, the role of autophagy in cancer is controversial and depends on genotype and tumor stage development [139]. Many clinical trials examined the synergistic effects of the addition of HCQ to conventional chemotherapic drugs, finding that the role of autophagy is complex and is influenced by several factors. Depending on genetic concomitant alterations, autophagy may possess both pro-tumorigenic and tumor-suppressive roles. It has been confirmed in murine models of pancreatic ductal adenocarcinoma, a type of cancer with a high mortality rate, due to its refractoriness to therapies. Mice presenting activated oncogenic KRAS and normal expression of p53 oncosuppressor experienced a critical regression of tumor developing under HCQ (60 mg/kg/day i.p.). By contrast, in those with a deficiency of p53, the inhibition of autophagy by HCQ increased the tumor progression, demonstrating that autophagy’s role in tumorigenesis is strictly related to the expression of p53 [140]. The expression of p53 is often altered in cancer, so as to be found mutated or absent in the 75% of pancreatic ductal adenocarcinomas [141]. This issue highlights the necessity to carefully evaluate the use of HCQ in certain tumor types. Different outcomes have been previously described, it has been found that inhibition of autophagy by HCQ might arise as a valuable adjuvant in pancreatic ductal adenocarcinoma chemotherapy, regardless of *p53* status [142,143]. Given the same doses and route of administration, these inconsistencies between the two reported studies could probably derive from the use of p53 homozygous and heterozygous models of mice, respectively. Regarding KRAS oncoprotein, its downstream pathway is one of the major players of pancreatic carcinogenesis. The inhibition of this pathway by cytotoxic drugs, as well as trametinib, is often associated with an increase in autophagy. For this reason, Drucker and Rosen [144] performed an off-label trial with an association of 400–1200 mg of HCQ and a constant dose of trametinib, observing a partial response with a general reduction of tumor lesion size, circulating tumor markers and cancer-associated pain.

In other cancer types, such as ovarian, prostatic, and human breast cancer, the anticancer or pro-tumorigenic effects of HCQ are determined by tumor stage. In the early stages of the disease, the inhibition of autophagy results in an inhibition of tumorigenesis, while in the advanced phase, it enhances cancer survival [145]. Then, it is important in assessing the contextual role of HCQ in cancer resistance mechanisms. Epirubicin in triple-negative breast cancer therapy often lost efficacy, due to chemoresistance acquiring. It has been shown that this cytotoxic agent induced autophagic flux, increasing cancer cell survival. The combination with HCQ (120 mg/kg by i.p.), thanks to the anti-autophagic properties, significantly suppressed tumor growth by up to 50% with respect to the monotherapy [142]. In addition, estrogen receptor-positive breast cancers developed resistance to treatment with tamoxifen, due to the enhancement of autophagy. The coadministration of HCQ (1–2 mg/day/mice in drinking water) restored the susceptibility of cancer cells to tamoxifen [146]. In mice with thyroid gland xenograft carcinoma, HCQ (150 mg/kg/day p.o. for two weeks) did not provide significant results on tumor growth, while the combination of HCQ with the chemotherapic agent vemurafenib potentiated the anticancer properties of both drugs [147]. Similarly, the two weeks coadministration of HCQ (65 mg/kg) and CCI-779 resulted in a synergism that significantly enhanced their in vivo activity against melanoma tumor growth, in terms of tumor size, with respect to their single treatment [148]. HCQ was revealed also to be active against chemoresistant lung cancer. In this type of cancer, the hypoxic conditions led to lesser susceptibility of cancer cells towards lymphocyte T-mediated cytolysis, thanks to the activation of autophagy. HCQ intake, at doses of 30 mg/kg/day i.p. for 10 days, sensitized tumor cells to lysis and allowed, together with conventional treatment, the eradication of the tumor [149].

Together with autophagy, glycolysis plays a pivotal role in satisfying the increased energetic demand. The dual targeting of the processes may provide a new therapeutic approach in cancer cells. Emonet, et al. [150] performed a randomized preclinical study on Earlic ascites hepatoma-bearing mice, showing that the coadministration of HCQ (60 mg/kg i.p.) and the antiglycolytic inhibitor 3-bromopyruvate possessed a synergistic effect on tumor growth inhibition. Moreover, this treatment is associated with an improvement of oxidative status in hepatic tissue, with a decrement in the number of cancer cells, without affecting the total cell count [151].

Resistance mechanisms also involved alterations in *β*-Cell Lymphoma (Bcl) Bcl-2 and Bcl-xL and anti-apoptotic gene expression. To evaluate the validity of the dual approach, targeting both apoptosis and autophagy, HCQ (50 mg/kg i.p.) and an apoptosis inhibitor, ABT-737, were administered to prostatic cancer xenograft mice for 15 days. Tumor growth was significantly suppressed by a combination of drugs, with respect to HCQ or ABT-737 alone [152]. In the same way, Fenollar, et al. [153] demonstrated the efficacy of Obatoclax, a pan-Bcl-2 inhibitor, used in association with HCQ (3–60 mg/kg) or conventional in neuroblastoma-bearing mice. Positive outcomes regarded the diminution of tumor size and the complete absence of metastases in cotreated mice with respect to Obatoclax alone or with respect to control [154]. Apoptosis is also at the base of the anticancer activity of interferon-alpha, but the cancer treatment with this drug alone often leads to chemoresistance. It has been demonstrated that autophagy is in the main responsible for chemoresistance, thus the combination of interferon-alpha with inhibitors of autophagic flux may be a useful therapeutic approach. In 30 xenograft mice with head and neck squamous carcinoma, the combination of interferon-alpha with HCQ (60 mg/kg/day i.g.) and wortmannin synergistically promoted apoptosis and inhibited tumor growth [155]. In a similar fashion, Le Goff, et al. [156] investigated the potential synergic role of HCQ (30 mg/kg) in enhancing the anticancer activity of melatonin on tongue squamous cell carcinoma mouse models. The anticancer activity of melatonin depends on its pro-apoptotic effects. Nevertheless, this activity is accompanied by a pro-autophagic activity that caused chemoresistance. The coadministration of the autophagy inhibitor HCQ strongly enhanced melatonin anticancer efficacy, resulting in a smaller tumor size and weight. The effect of inhibition of autophagy on tumor growth may be enhanced if the inhibition of autophagic flux occurs when the process of autophagy is quite completed. This hypothesis has been evaluated by Brönnimann, et al. [157], administering by intravenously TAT–Beclin 1 peptide and HCQ (65 mg/kg) in murine models of breast cancer. Initially, the first agent induced the autophagic flux with the production of autophagosomes, while in the final phase of the process, the second stopped the autophagy by deacidification of lysosomes, causing the accumulation of autophagic vesicles and tumor death. HCQ was administered as HCQ-loaded liposomes, to modulate the onset of autophagy inhibition [158]. This formulation allows us to overcome the limits of HCQ usage, related to the high doses required, which is often unachievable in humans. Relatively high doses of HCQ were loaded in nanoparticles, together with the cytotoxic drug chlorambucil, demonstrating it to be safe and efficient in killing leukemia/lymphoma cancer cells in a human-mouse model of Burkitt’s lymphoma. Eight injections of nanoparticles containing 400 mg of HCQ and chlorambucil led to the overall survival of mice. These concentrations of free drugs are inapplicable, due to their high toxicity [159]. As demonstrated by Naso, Wong, Wong, Chen and Hoang [72], HCQ liposomes (60 mg/kg), together with a pH-sensitive targeting peptide that delivered HCQ into the tumor cells and lysosomes, enhanced the chemotherapic effect of conventional anticancer drug doxorubicin in animal models of melanoma. Likewise, Vayssade, et al. [160] conceived a nanogel (CA4-FeAlg/HCQ) for co-addressing vascular blocker CA4 and anti-autophagic agent HCQ (30 mg/kg) in tumor blood vessels, to synergistically treat A549 lung cancer in mice. Firstly, the release of CA4 exerted anti-angiogenic effects in the vascular site, then FeAlg/HCQ were released into small nanogels and entered in the tumor, where HCQ inhibited autophagy and iron generated ROS with a synergic antitumor effect [161]. Similarly, De Jong, et al. [162] evaluated the response of an animal model of pancreatic cancer to HCQ (5 mg/kg) and paclitaxel administration, loaded in liposomes, modified with an acid environmental sensitive peptide that is responsible for site-specific delivery. Tumor weight, together with the number of liver metastases, was significantly reduced. The administration of HCQ is associated with the inhibition of autophagy and the reduction of IL-6 that is responsible for cross-talking between cancer cells and fibroblasts. All these events avoided the formation of stroma fibrosis, allowing paclitaxel to easily reach the tumor site [104]. The synergism results are essential for HCQ activity in pancreatic cancer. In monotherapy, indeed, HCQ (800–1200 mg/day) did not achieve significant autophagy. This resulted in negligible therapeutic effects in patients with already-treated metastatic pancreatic cancer, of which only 10% were without the progressive disease after two months of therapy [163].

Therefore, the use of modified formulations, such as liposomes, nanogels, etc., may be a precious tools for drug codelivery at the tumor site, enhancing efficacy and reducing side effects. Moreover, the availability of HCQ in those formulations encouraged the use of this molecule in brain tumors, as this formulation highly improved the penetration of this drug in the brain–blood barrier. The co-encapsulation of HCQ with a tyrosine kinase inhibitor, ZD6474, exhibited a synergistic effect, increasing the survival of glioma-bearing mice by two-times with respect to free ZD6474. Those synergistic effects are attributable to significant inhibition of autophagy exerted by HCQ and might provide a valuable therapeutic tool in glioma treatment [164]. 

The anticancer effect of free HCQ at 200–800 mg/day p.o. has been evaluated in two similar clinical trials on glioblastoma patients in concomitant temozolomide drugs and radiotherapy. Although a dose-dependent significant increase of autophagy markers, no significant effects on tumor suppression were recorded in both studies, as the dose-limiting toxicity was not allowed to achieve higher doses of HCQ [162,165]. The maximum tolerated dose is the dose over which at least one patient from six experienced dose-limiting toxicity, including myelosuppression, anorexia, fatigue, or nausea. Moreover, it has been proved that HCQ severely altered the organization of the Golgi apparatus and the endolysosomal system in C57BL/6JolaHsd mice under 60 mg/kg/day of HCQ i.p. [166]. However, different from Rosenfeld’s studies, no maximum tolerated dose was reached for HCQ in combination with chemotherapic temsirolimus, allowing us to perform a dose-escalation study on 27 patients with solid tumors and 12 with a melanoma diagnosis. In both cases, the standard intravenous dose of temsirolimus with 1200 mg of oral HCQ was considered safe and tolerated and inhibited tumor growth [167]. The same authors further assessed the HCQ anticancer properties and dose-limiting toxicity on 40 patients with metastatic melanoma, by administering a dose intense regimen of temozolomide and escalating doses of HCQ (200–1200 mg/day p.o.). Patients well tolerated the treatment, showing a positive response in the 14% of cases and stability of disease in 27%, due to the modulation of autophagy. No maximum tolerated dose was reached, although common toxicities were manifested [168]. According to the results of Rangwala, a phase I trial on 25 patients with myeloma demonstrated that the recommended dose of HCQ for a phase II trial is 600 mg twice a day. Among eligible patients, 14% experienced a very good response, 14% minor responses, and 45% a period of stability in the disease when the association of HCQ and bortezomib were provided. The synergic effect on myeloma was probably due to the combination of inhibition of HCQ on autophagy and bortezomib on proteasomal degradation, leading to the accumulation of misfolded proteins and autophagic vacuoles in cancer cells [169]. Likewise, doses of 600 mg of HCQ twice a day are not associated with toxicity and its usage as adjuvant therapy with everolimus was well tolerated and produced disease control in 67% of the metastatic clear-cell renal cell carcinoma patients and achieved the rate of six month progression-free survival in 45% of patients [170].

#### 2.4.4. Bacterial Infections

HCQ is known to exert an antibacterial effect through the alkalinization of infected organelles, inhibiting bacterial replication. In clinical practice, HCQ is not used in monotherapy but in combination with antibiotics, like doxycycline, to improve its bactericidal effects on two main bacteria: *Coxiella burnetii* and *Tropheryma whipplei*.

*C. burnetii* is an obligate intraleukocytic Gram-negative bacterium responsible for query fever (Q fever). The infection is mainly caused by direct contact with infected animals, although cases of human transmission have also been described. Q fever diagnosis is primally founded on serological examination and based on a different evolution, acute and chronic infection can be distinguished. In 50% of cases, the acute phase is asymptomatic, but when the acute phase is symptomatic, it is characterized by a febrile illness, myalgia, headache, chills, atypical hepatitis, and pneumonia [122,123]. Approximately 2–5% of *C. burnetii* infections can develop into the chronic phase, leading to endocarditis and vascular infection. The risk of developing chronic fever is higher in patients with pre-existing vascular disorders or valvulopathies [123,124]. *C. burnetii* is known to replicate in an intracellular phagolysosome with a pH range of 4–5. However, at this pH, antibiotics, like doxycycline (DXC), exert only a bacteriostatic activity. Therefore, a combination of DXC with a lysosomotropic agent, such as HCQ, was suggested. In fact, HCQ was shown to increase the phagolysosomal compartment’s pH by improving the bactericidal activity of doxycycline [125,126]. The first successful results concerning the treatment of Q fever endocarditis combined with DXC and HCQ date back to 1993 [127]. These results were later confirmed by a case report of a young infected girl, where the treatment with 200 mg/day of DXC and 600 mg/day of HCQ led to a reduction in serum *C. burnetii* antibodies within 48 h [128]. Furthermore, in a 1999 clinical study, the administration of 100 mg DXC twice daily plus 200 mg HCQ three times daily for at least 18 months led to a short duration of therapy and a reduction in recurrences compared to alternative treatments including DXC plus 200 mg ofloxacin three times daily [129]. Since this moment, all infected subjects have been treated with DXC plus HCQ, as demonstrated by several case reports where this regimen results in an improvement of *C. burnetii*-related disease [130,131,132,133,134,135,139,140]. Furthermore, in patients with valvulopathy and diagnosticated acute Q fever (serologic criteria of a phase II IgG titer ≥ 200 and a phase II IgM titer ≥ 50) the administration as prophylaxis of DCX plus HCQ for at least 12 months resulted to be efficient in preventing Q fever endocarditis. Contrarily, shorter regimes are associated with a failure of antibiotics prophylaxis [141]. When Q fever endocarditis occurs, the optimal treatment duration with DXC and HCQ seems to be 18 months for native valve patients and 24 months for subjects with prosthetic valves [142]. This duration should only be extended in the absence of favorable serological results. However, long-term treatment with DXC and HCQ is not without important complications, since both can cause photosensitivity [144], abnormal weight gain [145], severe erythroderma, and impaired visual field [142]. Besides, it can be said that while the acute phase of the infection can be treated with only 200 mg/day DXC, the chronic phase is more difficult to treat and therapy with 100 mg DXC twice daily with 200 mg HCQ three times daily for 18–24 months was recommended [146]. Serological titers are used to follow the disease and determine the duration of therapy.

On the other hand, *T. whipplei* is a Gram-positive bacterium responsible for Whipple’s disease. The natural niche of *T. whipplei* is the human intestine since, in the intestinal mucosa, the bacterium is taken by macrophages, where it replicates [147]. This bacterial infection is primally characterized by digestive tract disorders such as diarrhea (75% of cases), malabsorption, and weight loss (80–90% of cases). Joint disease may appear more than six years before the diagnosis and occur in more than 80% of patients [148]. Furthermore, neurological and cardiac disorders can also be frequently associated with Whipple’s disease. For years the standard treatment for *T. whipplei* has included a combination of trimethoprim and sulfamethoxazole; however, relapses were not uncommon [149,150]. Later, in vitro studies, demonstrated that trimethoprim was inactive on this bacterium [154], while sulfamethoxazole induced bacterial resistance, making the co-administration completely ineffective [154,156]. Based on the good results of treating *C. burnetii* infections, it was decided to test in vitro the association DCX/HCQ on *T. whipplei,* obtaining good results [154]. DCX/HCQ efficacy on *T. whipplei* diseases was demonstrated in a clinical trial dated 2014. This study showed that the administration of 200 mg/day DCX and 600 mg/day HCQ to 13 patients results in better outcomes (0/13 failures) even after 1 year of treatment, compared to standard antibiotics regimens [155]. To date, several case reports available in the literature supported a therapy consisting of a combination of HCQ (600 mg/day) and DCX (200 mg/day) for a lifetime or at least one year, followed by a maintenance dosage of DXC used alone [156,157,158]. In some cases, prophylaxis of intravenous ceftriaxone (2g/day) for the first two weeks followed by HCQ/DXC for at least 12–18 months has been recommended [72,159,160,161].

**Figure 8:**
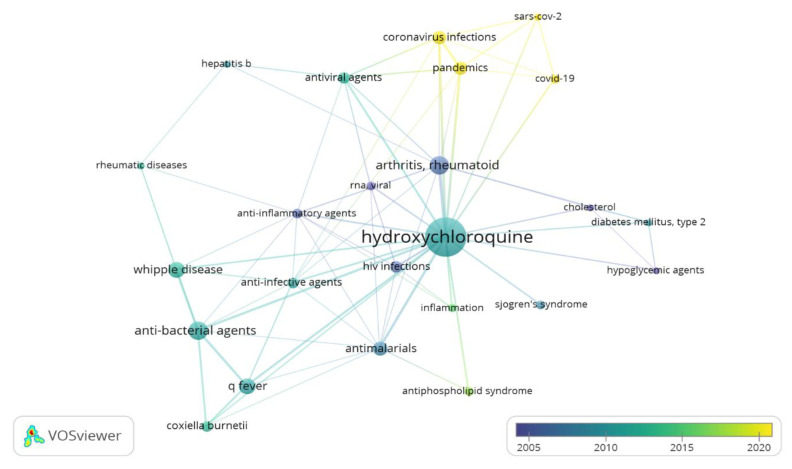
Bubble map visualizing items from articles included in the review. A total of 24 items representing the different fields of action of HCQ have been grouped into clusters, based on their relatedness. The distance between the two terms represents the relatedness of the terms. Generally, the smaller the distance between two terms, the stronger the relationship of the terms to each other. Two items are closer to each other if they co-occurred more frequently in the evaluated publications. The item size indicates the words’ appearance frequency (multiple appearances in a single manuscript count as one). As explained in the legend, the time colors indicate the research focus over the years.

Although HCQ was revealed to be effective against bacterial infections, in the last few years, in light of the current epidemiological situation, the research attention has shifted toward HCQ application as an antiviral agent, as it could be seen in the bubble map (Figure 8). This visual map is obtained by VOSviewer software, analyzing recurring items from all keywords [171].

## 3. Materials and Methods

### 3.1. Search Strategy

According to Preferred Reporting Items for Systematic Reviews and Meta-Analyses (PRISMA) guidelines, a systematic literature search was performed in May 2020 and included all reports published to August 2020. The search was performed on specialized databases (PubMed and Scopus) using different combinations of HCQ and the following keywords: history, discovery, ethnomedical, synthesis, chemical structure, SAR, RAS, biological activity, approved biological activities, antiviral, antiviral mechanism, COVID-19, Q fever, Whipple’s disease, synergistic effects, synergic effects, toxicological effects, toxic effects, toxicity, animal model and antiviral activity, clinical study, preclinical study. We did not request full-text to investigators if not available and we did not try to find unpublished data. 

### 3.2. Study Selection

The manuscript selection was based on the inclusion criteria: preclinical (in vivo) and clinical studies involving the use of HCQ and combinations, only articles published in English and containing keywords in the title or in the abstract were selected. Other review articles, meta-analysis, retrospective studies, abstracts, conferences, editorials, letters, conference proceedings, manuscripts without full text available or articles that did not meet the inclusion criteria were not included in this systematic review. For selecting the sources, three independent investigators (I.F., F.L., and M.P.) first selected the articles according to the title and abstract and then by analyzing the full-texts. In cases of non-consensus, authors tried to resolve any disagreements by discussion or, if necessary, two more independent reviewers were consulted (L.M. and N.D.T.). The selected articles were carefully reviewed with the aim of identify or exclude the manuscripts that did not fit the criteria described above. Additional papers were added to this review after the analysis of the bibliography from the included articles.

### 3.3. Data extraction

Data were collected and examined by the authors and information from the selected manuscripts on HCQ, as well as study design, experimental models, general mechanisms implicated in antiviral and biological activities, major outcomes doses or concentrations, and route of administration were extracted.

### 3.4. Methodological Quality Assessment

The risk of bias and the quality of the preclinical and clinical investigations were assessed independently by the authors, using a checklist adapted from *Cochrane Handbook for Systematic Reviews of Interventions*, specifically adjusted for animal intervention study (SYRCLE’s) [171,172] and clinical trials [97]. The evaluation of the selected studies’ methodological quality was based on the presence or absence of information regarding the main objectives and findings, randomization of the treatment allocation, blinded drug administration, blinded outcome assessment and outcome measurements, as reported in Table 3 and Table 4. Only studies that reported a positive judgment in all considered parameters were assessed to be of a higher methodological quality. In contrast, the studies that did not wholly fulfil the criteria were included in the medium risk of bias, while those that completely lacked this information were deemed to be at high risk of bias.

## 4. Conclusions

The bubble map of Figure 8 summarizes the pleiotropic activity of HCQ evaluated in this review. As highlighted by the colorimetric variation, the research, in the early 2000s, has been focused on the application of HCQ as an antimalarial drug (blue color). In contrast, in the last few years, scientists have moved their attention to the influence of HCQ on many pathways involved in inflammation, infections, autoimmune diseases, cardiovascular pathologies, and diabetes (blue to green color). Finally, in the last months, it is evident that the rapid spread of the COVID-19 pandemic has led to the revaluation of HCQ in viral infections (yellow color). However, the analysis of currently available clinical studies showed that the administration of HCQ to prevent and cure COVID-19 infection is questionable, since results from clinical trials are contrasting, and the last data did not support the use of HCQ for the treatment and prevention of COVID-19 disease. Despite these results, HCQ is considered to be a safe drug since it is generally better tolerated than other 4-aminoquinolines, such as CQ. Hence, nowadays, HCQ arises as a first-line treatment in managing autoimmune diseases such as rheumatoid arthritis, lupus erythematosus, and Sjögren syndrome, mainly in association with methotrexate or corticosteroids, showing a synergistic effect on disease control. It also improves glucose and lipid homeostasis and revealed significant antibacterial activity in combination with DXC. To better characterize HCQ activity, computational models should be useful for targeting and docking the molecular features responsible for its mechanism of action. Based on this work, it should be possible to hypothesize future applications of HCQ in medical therapy.

## Figures and Tables

**Table 1:** Antiviral effects of HCQ, outcomes.

Author(Year)	Study TypePopulation	DosageTime	Outcomes	Adverse Events Noted	Limitation of the Study
HIV-1					
Sperber, et al. (1995) [17]	Randomized, double-blind, placebo-controlled clinical trial 40 asymptomatic HIV-1 infected patients	HCQ group - > 800 mg/day Control group - > placebo 8 weeks	Total HIV-1 RNA plasma levels significantly decreased in the HCQ group (range, 98 to 2517 cpm; mean, 168 ± 144 cpm *vs*. 311 ± 331 cpm; *p* = 0.022). CD4^+^ T cells percentage remained stable in HCQ group (18.1 ± 9.2% before treatment *vs*. 18.6 ± 10.5% after treatment) Absolute CD4^+^ has not reported significant changes in both groups IL-6 and IgG levels decrease in HCQ group (14.3 ± 13.5 U/mL *vs*. 12.0 f 16.7 U/mL; *p =* 0.023 and 2563 ± 1352 mg/mL *vs*. 2307 ± 1372 mg/dL; *p =* 0.032, respectively)	Not reported.	Small sample-size. All of the patients were asymptomatic with a low viral load. A short period of study time.
Sperber, et al. (1997) [18]	Randomized, placebo-controlled clinical trial 72 asymptomatic HIV-1 infected patients	800 mg/d HCQ (*n* = 35) 500 mg/d ZDV (*n* = 37) 16 weeks	After 16 weeks total plasma HIV-1 RNA levels were reduced in both ZDV group (42.709 ± 33.050 *vs*. 11.228 ± 7459 copies/mL; *p* = 0.001) and HCQ group (39.456 ± 31.000 *vs*. 16.434 ± 11.373 copies/mL; *p* = 0.02). No significant change occurred in CD4^+^ cells Only in HCQ group it was a reduction in the levels of IL-6 (12.4 ± 12.9 *vs*. 6.3 ± 5.4 U/nL; *p =* 0.03) and Ig-G (1453 ± 453 *vs*. 395 ± 544 mg/dL; *p =* 0.02)	Not reported.	Small sample-size. All of the patients were asymptomatic.
Paton, et al. (2002) [19]	non-comparative clinical study 22 asymptomatic HIV-1 infected patients	HCQ (200 mg) + hydroxyurea (500 mg) + didanosine (125–200 mg), taken twice daily. 48 weeks	In the 12th week there was a significant reduction of 1.3 log_10_ in viral load and an increase in CD4^+^ percentage by mean 4.3%. These values were maintained until the 48th week.	Not reported.	Small sample-size. This is a non-comparative design pilot study which not allow determining the contribution made by HCQ alone to the overall decrease in viral load obtained by the combination.
Paton, et al. (2005) [20]	open-label, noncomparative stud 17 HIV-1 infected patients	HCQ (200 mg) + hydroxyurea (500 mg) + didanosine (125–200 mg), taken twice daily. 144 weeks	Mean viral load was reduced by 1.6 log_10_ copies/mL below baseline (*p* = 0.001) CD4^+^ cell counts were significantly increased by a mean of 3.3 ± 6.9%, *p* = 0.095 at 144th week. CD8 cells percentage was reduced by 11.5 ± 14% per 48th week (*p* = 0.005) and remained around 10% until the 144th week	Not reported.	Small sample-size. Absence of a control group.
Aguirre-Cruz, et al. (2010) [21]	Randomized clinical study 8 HIV-infected adults with adenoid hypertrophy were included.	Group A - > 400 mg/day Group B - > 800 mg/day 8 days	HCQ main concentration was significantly higher in at than in plasma	Not reported.	
González-Hernández, et al. (2014) [22]	In vivo on rabbit model	Subcutaneous HCQ injection of 15 mg/kg of body weight.	HCQ had a higher affinity for lymphoid tissues than for blood.	Not reported.	
Piconi, et al. (2011) [23]	Prospective noncomparative Study 20 HIV-infected immunologic non-responders	400 mg/day HCQ 6 months	After 6 months, there was an increase in CD4^+^ T-cells percentage; a reduction of activation/proliferation in CD4^+^ T-cells (Ki67^+^) and CD14^+^ cells (CD69^+^); a decrease of plasma LPS levels; a downregulation of TLR-7/8 expression.	One patient reported maculopapular exanthema after 10 days of treatment.	Small sample-size. Patients were taking antiretroviral drugs during the treatment with HCQ.
Paton, et al. (2015) [24]	Randomized, double-blind, placebo-controlled trial 83 asymptomatic HIV-1 infected patients	400 mg/day HCQ or placebo 48 weeks	At 48th in HCQ group is revealed a faster decline of CD4^+^ T-cell counts; no change in activation/proliferation levels in CD8^+^ and CD4^+^ T-cells; no change in IL-6 levels; an increase in viral load.	Patients in the HCQ group reported influenza-like illness compared with the placebo group (29% *vs*. 10%; *p* = 0.03).	Small sample-size.
Chen, et al. (2018) [25]	In vivo on a rabbit model	Intravaginal implant designed to release an HCQ concentration above 4.34 µg/mL but below 21.7 µg/mL 6 days	After 6 days, there was seen an improvement of mucosal epithelial integrity, a reduction in submucosal immune cell recruitment, a decrease of gene expression and T cell activation marker protein, and a minimization of key pro-inflammatory mediators activation.	Not reported.	No clinical study has been designed to test the effectiveness of HCQ in preventing HIV infection
**Chikungunya Virus**					
Padmakumar, et al. (2009) [26]	Prospective, randomized, parallel-group study 120 patients in the acute phase of CHIKV infection	Group A -> 200 mg/day ACF Group B -> ACF + 400 mg/day HCQ Group C -> ACF + 10 mg/day PRD Group D -> ACF + HCQ + PDR	HCQ did not confer any additional benefit in the treatment of the early stages of chikungunya.	Not reported.	The duration of the study can be considered as a limitation with respect to the efficacy assessment of HCQ, which is a slow-acting drug.
Bouquillar, et al. (2018) [27]	Multicenter study 39 patients with chronic CHIKV infection	400 mg/day HCQ 3 months	After three months of treatment, evidence of synovitis was disappeared in 10 of 20 subjects (50%) with swollen joins while complete remission was verified in 5 patients (19.2%)	In four subjects, the treatment was interrupted due to the onset of side effects such as nausea, stomatitis, rash, and headache.	Small sample-size.
Ravindran, et al. (2017) [28]	Randomized controlled open-label study 72 patients with chronic CHIKV infection	400 mg/day HCQ (*n* = 35) 15 mg/day MTX, 1g/day sulfasalazine, and 400 mg/day HCQ (*n* = 37) 34 weeks	At the end of the 24th week, only the combination of drugs improved disease activity (mean ± SD DAS28; 3.39 ± 0.87 *vs*. 4.74 ± 0.65, *p* < 0.0001) and reduces disability (mean ± SD HAQ; 1.4 ± 0.31 *vs*. 1.8 ± 80.47, *p* < 0.0001) and pain (mean ± SD VAS 46 ± 6.13 *vs*. 60.8 ± 11.6, *p* < 0.0001).	In the combination group, one patient withdrew due to nausea.	It is not a blinded study and so the bias in reporting improvement could be present.
Pandaya S. (2008) [29]	Uncontrolled clinical study 305 patients with chronic CHIKV infection	15–20 mg/weekly MTX + 400 mg/day HCQ 16 weeks	At 16th week a reduction in ACR score was shown	Not reported	There is not a control group. Only 114 subjects completed the study. It is not a blinded study and so the bias in reporting improvement could be present.
**Flavivirus**					
Helal, et al. (2016) [30]	Prospective, randomized, controlled, interventional, single-blind study 120 patients affected by hepatitis C virus	Group 1 -> SOC (160 µg pegylated interferon subcutaneously and 1000–12000 mg/day ribavirin orally) Group 2 -> SOC + 200 mg/day HCQ 12 weeks	HCQ + SOC group showed a high virological response compared to control group [54/60 (90%) *vs*. 43/60 (71.7%); *p* = 0.011] and a normalization of ALT levels.	Both groups showed symptoms such as headache, fatigue, influenza-like illness, and gastrointestinal disturbance.	A short period of study time. There was a lack of the rapid virological response (RVR) assessment of defined as HCV RNA negativity at week 4 of treatment.
Cao, et al. (2017) [31]	In vivo study on pregnant mice infected with ZIKV	40 mg/kg/day HCQ	HCQ attenuated placental and fetal ZIKV infection and ameliorated adverse placental and fetal outcomes	Not reported.	No clinical study has been designed to test the effectiveness of HCQ in preventing ZIKV infection.
**COVID-19**					
Chen et al. (2020) [6]	Randomized, parallel-group clinical trial 62 patients suffering from COVID-19	HCQ group -> 400 mg/day HCQ Control group -> SOC Day 5	Body temperature recovery time in the HCQ group was shorter than the control group (2.2 *vs*. 3.2 days, *p* = 0.0008). Cough remission time was significantly decreased in the HCQ group (2.0 *vs*. 3.1 days, *p* = 0.0016). Improvement of pneumonia in HCQ group (80.6% *vs*. 54.8%) Pneumonia absorption in HCQ group (61.3%)	One patient developed a rash. One patient reported a headache.	Small sample-size. Detail about antiviral and antibacterial agents used in the control group are not available.
Gautret et al. (2020) [32]	Open-label non-randomized clinical trial 36 patients	HCQ group -> 600 mg/day HCQ (*n* = 14); 600 mg/day HCQ +500 mg AZM on day 1 followed by 250 mg/day for 4 days (*n* = 6) Control group (*n* = 16) Day 10	On day 6, 70% of HCQ-treated patients were virologically cured comparing to 12.5% in the control group On day 6, 100% of HCQ+AZM treated patients are virologically cured comparing to 57.1% in the HCQ group and 12.5% in the control group.	Gastrointestinal side effects in one patient of HCQ group. One patient of the HCQ group died on day 3 although he was PCR-negative on day 2.	Small sample-size. Dropout of six patients from HCQ group. Data available up to 6 days despite the planned 10 days. Details about control group treatment are not available.
Gautret, et al. (2020) [33]	Uncontrolled, non-comparative, observational study 80 mildly infected patients	600 mg/day HCQ per 10 days + 500 mg AZM on day 1 followed by 250 mg/day for 4 days For patients with pneumonia and NEWS score ≥ 5 ceftriaxone was added to HCQ/AZM treatment	On day 7, nasopharyngeal viral load tested by qPCR was negative for 83% of patients and for 93% of patients at day 8. At day 5 in 97.5% of patients, virus cultures of the respiratory sample were negative. After 10 days only 2 patients were contagious.	One patient died. Six patients had GI side effects (2 nausea or vomiting and 4 diarrhea) One patient had blurred vision.	Six patients from previous trials by Gautret et al. were also included in this study. No analytical approach has been made to take into account possible factors of confusion, including in particular the severity of the disease.
Molina et al. (2020) [34]	Prospective, non-comparative study 11 severe COVID-19 infected patients	600 mg/day HCQ per 10 days + 500 mg AZM on day 1 followed by 250 mg/day for 4 days	On day 5 two patients were transferred to the ICU. At days 5 to 6, after treatment initiation 8 of 10 patients were still positive for SARS-CoV2 RNA.	One patient died. One patient discontinued the treatments due to QT interval prolongation.	Small sample size, 8 of 11 had comorbidities associated with poor outcomes.
Tang et al. (2020) [35]	Multicenter, open-label, randomized controlled trial 150 mild/moderate or severe COVID-19 infected patients	HCQ group -> SOC+ HCQ (200 mg daily for three days followed by a maintained dose of 800 mg daily) Control group-> SOC 2 for mild/moderate patients and 3 weeks for severe patients	Within 28 days of treatment, the probability of negative conversion of SARS-CoV-2 was 85.4% (95% CI 73.8% to 93.8%) in the HCQ + SOC group and 81.3% (95% CI 71.2% to 89.6%) in the SOC group. No significant differences in the median time to negative conversion were found between the HCQ + SOC group (8 days, 95% CI 5 to 10 days) and SOC group (7 days, 95% CI 5 to 8 days). No difference in PCR negativity was found between two groups at day 4, 7, 10, 14, or 21. No significant differences in the meantime of clinical symptom alleviation were found between the two groups (19 days for HCQ + SOC *vs*. 21 days for SOC)	Adverse events noted in 30% of the HCQ group compared to 8.8% of control group The most common adverse effect was diarrhea (10%). One patient had blurred vision.	The study is only based on the virus-negative conversion.
Abd-Elsalam, et al. (2020) [36]	Multicenter, randomized controlled trial 194 COVID-19 infected patients	HCQ group -> SOC+ HCQ (400 mg twice daily, on day 1, followed by 200 mg tablets twice daily) Control group -> SOC 4 weeks of treatment	There was no significant difference between the two groups regarding any laboratory parameters or the baseline characteristics. Four patients (4.1%) in the HCQ group and 5 (5.2%) patients in the control group needed mechanical ventilation (*p* = 0.75). There were no differences in the overall mortality between the two groups, as six patients (6.2%) died in the HCQ group and five (5.2%) died in the control group (*p* = 0.77).	Not reported.	Small sample size, which was not adequately powered for survival endpoints. Lack of long-term follow-up.
Skipper, et al. (2020) [37]	Randomized, double-blind, placebo-controlled trial 491 symptomatic, non-hospitalized adult patients with early or mild COVID-19	HCQ group -> HCQ 800 mg once, followed by 600 mg in 6 to 8 h, then 600 mg daily for 4 more days Control group-> masked placebo 14 weeks of treatment	HCQ did not reduce symptom severity when compared with placebo in non-hospitalized early/mild COVID-19 patients (difference in symptom severity: relative, 12%; absolute, −0.27 points (95% CI, −0.61 to 0.07 points); *p* = 0.117)	With HCQ, the most commonly reported adverse effect was related to gastrointestinal symptoms: 31% (66 of 212) of participants reported upset stomach or nausea, and 24% (50 of 212) reported abdominal pain, vomiting, or diarrhea.	Lack of confirmed SARS-CoV-2 infection in all participants. The use of epidemiologic linkage to enroll symptomatic persons.
Mahévas, et al. (2020) [38]	No-randomize clinical study 181 COVID-19 infected patients	HCQ group -> 600 mg/day for 5 days (*n* = 84) within 48 h of admission to hospital Control group (*n* = 97)	Within day 7: 20.2% infected patients of the HCQ group and 22.1% in the control group died or were transferred to the ICU; 27.4% of the HCQ group and 24.4% of the no-HCQ group shown acute respiratory distress; On day 7 the percentage of death was similar in both HCQ and control group (2.8 *vs*. 4.8%, 3 *vs*. 4 events)	7 patients of the HCQ group showed QT interval prolongation. One patient presented first-degree atrioventricular block after 2 days of HCQ administration.	The study was not randomized. Potential unmeasured confounders may bias the results.
Mahévas, et al. (2020) [39]	Observational comparative study 181 severe COVID-19 infected patients	HCQ group -> 600 mg/day (*n* = 92) Control group -> SOC (*n* = 89)	On day 21: Overall survival was 89% in the HCQ group and 91% in the control group; survival without acute respiratory distress syndrome was 69% in the HCQ group and 74% in the control group; patients who had been weaned from oxygen was 82% in the HCQ group and 76% in the control group.	7 patients of HCQ group showed QT interval prolongation One patient presented first-degree atrioventricular block after 2 days of HCQ administration.	Treatment was not randomly assigned and potential unmeasured confounders could bias the results. Patients from previous trials by Mahévas et al. were also included in this study.
Lee, et al. (2020) [40]	Single-center clinical study 211 individuals exposed to COVID-19	400 mg day of HCQ as post-exposure prophylaxis 14 days	At the end 14 days of quarantine, there was negative follow-up PRC tests.	The most common side effects were diarrhea or loose stool (9%), skin rash (4.3%), gastrointestinal upset (0.95%) and, bradycardia (0.95%). In 5 patients (2.7%) post-exposure prophylaxis was discontinued due to bradycardia (2), gastrointestinal upset (2), and the need for fasting (1).	There was not a control group and the study was carried out at a single center.
Boulware, et al. (2020) [41]	Randomized, double-blind, placebo-controlled clinical trial 821 asymptomatic participants	HCQ group: 800 mg once, followed by 600 mg in 6 to 8 h, then 600 mg daily for 4 additional days Placebo group	The incidence of new illness compatible with Covid-19 did not differ significantly between the HCQ group (49 of 414 (11.8%)) and the placebo group (58 of 407 (14.3%)); the absolute difference was −2.4 percentage points (95% confidence interval, −7.0 to 2.2; *p* = 0.35).	Nausea, loose stools, and abdominal discomfort were the main side effects. There were no intervention-related severe adverse reactions or cardiac arrhythmias.	Small sample-size
Maissonasse, et al. (2020) [40]	In vivo study on macaques	Different strategies of treatment were compared with placebo, including HCQ alone or in combination with AZM, administrated either before or after viral load	When HCQ was administrated as pre-exposure prophylaxis, it did not protect against infection acquisition. Neither HCQ nor HCQ + AZM had beneficial effects in improving viral infection’s symptoms.	Not reported.	

The abbreviations are for Hydroxychloroquine (HCQ), Zidovudine (ZDV), Aceclofenac (ACF), prednisolone (PRD), Methotrexate (MXT), Azithromycin (ZAM), Standard-of-care (SOC), Intensive Care Units (ICU), Chikungunya Virus (CHIKV), Zika virus (ZIKV).

**Table 2:** Main mechanisms of action underlying biological effects of HCQ.

Disease	Experimental Model	Dosage	Mechanisms of Action	References
Rheumatoid arthritis (RA)	Preclinical	40 mg/kg/day	↓neutrophil-derived oxidants ↓inflammation	[75]
	Clinical (randomized double-blind, placebo-controlled trial)	7 mg/kg/day	↓inflammation	[76]
	Clinical (comparative randomized double-blind trial)	200–400 mg/day	↓inflammation	[77,78]
RA-associated cardiovascular disease	Clinical	n.a.	↓IL-6 and leptin ↓dyslipidemia	[79]
	Clinical (cohort study)	6.5 mg/kg/day	↓triglycerides and LDL ↓dyslipidemia	[80]
	Clinical (randomized double-blind cross-over trial)	6.5 mg/kg/day	↓cholesterol and LDL ↓dyslipidemia	[81]
	Clinical (cross-sectional observational study)	200 mg/kg/day	↓fasting glucose	[82]
Systemic lupus erythematosus (SLE)	Clinical (randomized double-blind placebo-controlled trial)	100–400 mg/kg/day	↓inflammation ↓risk of exacerbations	[83]
	Clinical (long-term randomized study)	272 mg/day	↓inflammation ↓risk of exacerbations	[84]
	Clinical (case-control study)	6.5 mg/kg/day	↓inflammation ↑ survival	[85]
	Preclinical	100 mg/kg/day	↓Th17 response ↑Treg immunosuppressive effects	[86]
	Clinical (prospective cohort study)	400 mg/day	↓inflammatory markers	[87]
	Clinical (multiethnic US cohort)	n.a.	↓IFN-α	[88]
	Preclinical	4–40 mg/kg/day	↓ mast cells ↓ skin lesion	[89]
SLE-associated cardiovascular disease	Preclinical	10 mg/kg/day	↓ROS ↓endothelial damage	[90]
	Preclinical	3 mg/kg/day	↓ROS and nitric oxide ↓ endothelial damage	[91]
	Clinical	400 mg/day	↓triglycerides and LDL	[92]
	Clinical (cross-sectional study)	400 mg/day	↓ fasting glucose	[82]
SLE-associated pregnancy complications	Clinical (randomized double-blind)	n.a.	↓inflammation	[93]
	Clinical (prospective study)	6.5 mg/kg/day	↓inflammation ↓risk of exacerbations	[94]
Antiphospholipid syndrome	Preclinical	200 μg/day	↓inflammation ↓complement activation ↓placental abnormalities	[95]
	Clinical (case report)	400 mg/day	↓vascular thrombosis	[96]
	Preclinical	12 μg/g/day	↓endothelial damage ↓nitric oxide synthase	[97]
	Preclinical	20 mg/kg/day	↓endothelial damage ↓nitric oxide synthase	[98]
	Clinical (observational prospective study)	200 mg/day	↓thrombotic events in patients ↓soluble tissue factor levels.	[99]
Sjögren syndrome	Clinical	200 mg/day	↓inflammation ↓IgG and IgA	[100]
	Clinical (prospective study)	400 mg/day	↓xerostomia	[101]
	Clinical (prospective study)	6.5 mg/kg	↓eye dryness	[102]
	Preclinical	50 mg/kg/day	↓ xerostomia ↓ TGF-β↓inflammation	[103]
	Preclinical	60 mg/kg/day	↓inflammation ↓lymphocytic infiltration	[104]
Diabetes	Preclinical	80–120–160 mg/kg/day	↓blood glucose	[105]
	Preclinical	200 mg/kg/day	↓inflammatory markers ↑metabolic profile	[106]
	Clinical (randomized, double-blinded study)	2 × 300 mg/kg	↓glycated hemoglobin	[107]
	Clinical (open-label longitudinal study)	6.5 mg/kg/day	↓insulin resistance	[108]
	Clinical (randomized, double-blinded controlled trial)	6.5 mg/kg/day	↓insulin resistance	[109]
	Clinical (randomized, double-blinded trial)	400 mg/day	↑glycemic and lipidic profile	[110]
Cancer	Preclinical	50 mg/kg	↓tumor size ↓pro-tumorigenic and pro-inflammatory cytokines	[111]
Cardiovascular diseases	Preclinical	200 mg/kg	↓apoptosis in cardiomyocites	[112]
	Preclinical	200 mg/kg/day	↓triglycerides and LDL	[113]
	Preclinical	10 mg/kg/day	↓atherosclerosis ↓inflammation	[114]
Inflammatory bowel disease and colitis	Preclinical	30 mg/kg	↓inflammation	[74]
Pulmonary hypertension	Preclinical	50 mg/kg/day	↓inflammation	[115]
Endometriosis	Preclinical	60 mg/kg	↓inflammation ↓lesion number	[116]

LDL: low-density lipoproteins, Th17: effector lymphocyte T, Treg: regulatory lymphocyte T, IFN-α: type I interferon, ROS: radical oxygen species, Ig: immunoglobulin, TGF-β: transforming growth factor-β; n.a.= data not available.

**Table 3:** Checklist for assessment of the risk of bias in preclinical studies [171,172].

Checklist for Assessment of Risk of Bias in Preclinical Studies
Are the hypothesis and objective of the study clearly described?
Are the main outcomes to be measured clearly described?
Are the main findings of the study clearly described?
Are the samples size calculations reported?
Are the animals randomly housed during the experiment?
Are the investigators blinded from knowledge which treatment used?
Are the outcome assessors blinded?
Is the dose/route of administration of the HCQ properly reported?
Is the dose/route of administration of the drug in co-treatment properly reported?
Is the frequency of treatments adequately described?

**Table 4:** Checklist for assessment of risk of bias in clinical studies [97].

Checklist for Assessment of Risk of Bias in Preclinical Studies
Are the hypothesis and objective of the study clearly described?
Are the main outcomes to be measured clearly described?
Are the main findings of the study clearly described?
Are the samples size calculations reported?
Are the animals randomly housed during the experiment?
Are the investigators blinded from knowledge which treatment used?
Are the outcome assessors blinded?
Is the dose/route of administration of the HCQ properly reported?
Is the dose/route of administration of the drug in co-treatment properly reported?
Is the frequency of treatments adequately described?

## Abbreviations

ACF	Aceclofenac
At	Adenoid tissue
AZM	Azithromycin
Bcl	*β*-Cell Lymphoma
CHIKV	Chikungunya Virus
cpm	Counts Per Minute
COVID-19 or 2019-nCoV	new coronavirus delivery in 2019
CQ	Chloroquine
DMARDs	Disease-Modifying Antirheumatic Drugs
DXC	Doxycycline
ECG	Electrocardiogram
FGT	Female Genital Tract
HAART	Antiretroviral therapy
HCQ	Hydroxychloroquine
HIV	Human Immunodeficiency Virus
HOMA	Homeostatic Model Assessment
i.g.	Intragastrically
IgG	Immunoglobulin G
i.m.	Intramuscular injection
i.p.	Intraperitoneal injection
ICU	Intensive care units
IFN-α	Type I interferon
IL	Interleukin
LDL	Low-Density Lipoprotein
MXT	Methotrexate
NSAIDs	Non-steroidal anti-inflammatory drugs
p.o.	Per oral administration
PRD	Prednisolone
Q fever.	Query fever
RPE	Retinal Pigment Epithelium
ROS	Reactive Oxygen Species
SARS-CoV-2	Severe Acute Respiratory Syndrome caused by COVID-19
SOC	Standard-of-care
TGF-β	Transforming growth factor-β
TLR-4	Toll-like receptor 4
Tregs	T-regulatory cells
TNF-α	Tumor necrosis factors
VAS	Visual Analog Scale
ZDV	Zidovudine
ZIKV	Zika virus

## References

[1] Wallace D.J. (2001). Antimalarials-the “real” advance in lupus. Lupus.

[2] Wallace D.J. (1996). The history of antimalarials. Lupus.

[3] Bezati E., Wu X.-X., Quinn A.S., Taatjes D.J., Rand J.H. (2015). A new trick for an ancient drug: Quinine dissociates antiphospholipid immune complexes. Lupus.

[4] Coatney G.R. (1963). Pitfalls in a discovery: The chronicle of chloroquine. Am. J. Trop. Med. Hyg..

[5] Al-Bari M.A.A. (2015). Chloroquine analogues in drug discovery: New directions of uses, mechanisms of actions and toxic manifestations from malaria to multifarious diseases. J. Antimicrob. Chemoth..

[6] Chen Z., Hu J., Zhang Z., Jiang S., Han S., Yan D., Zhuang R., Hu B., Zhang Z. (2020). Efficacy of hydroxychloroquine in patients with COVID-19: Results of a randomized clinical trial. MedRxiv.

[7] Xu C., Zhu L., Chan T., Lu X., Shen W., Madigan M.C., Gillies M.C., Zhou F. (2016). Chloroquine and hydroxychloroquine are novel inhibitors of human organic anion transporting polypeptide 1A2. J. Pharm. Sci..

[8] Fantini J., Di Scala C., Chahinian H., Yahi N. (2020). Structural and molecular modeling studies reveal a new mechanism of action of chloroquine and hydroxychloroquine against SARS-CoV-2 infection. Int. J. Antimicrob. Agents.

[9] McLachlan A.J., Tett S.E., Cutler D.J., Day R.O. (1993). Disposition of the enantiomers of hydroxychloroquine in patients with rheumatoid arthritis following multiple doses of the racemate. Br. J. Clin. Pharmacol..

[10] Dongre V.G., Ghugare P.D., Karmuse P., Singh D., Jadhav A., Kumar A. (2009). Identification and characterization of process related impurities in chloroquine and hydroxychloroquine by LC/IT/MS, LC/TOF/MS and NMR. J. Pharm. Biomed. Anal..

[11] Tett S.E., Cutler D.J., Day R.O., Brown K.F. (1989). Bioavailability of hydroxychloroquine tablets in healthy volunteers. Br. J. Clin. Pharmacol..

[12] Al-Bari M.A.A.I. (2017). Targeting endosomal acidification by chloroquine analogs as a promising strategy for the treatment of emerging viral diseases. J. Pharmacol. Res. Perspect..

[13] Plantone D., Koudriavtseva T. (2018). Current and future use of chloroquine and hydroxychloroquine in infectious, immune, neoplastic, and neurological diseases: A mini-review. Clin. Drug Investig..

[14] Samuel C.E. (2001). Antiviral actions of interferons. Clin. Microbiol. Rev..

[15] Sperber K., Kalb T.H., Stecher V.J., Banerjee R., Mayer L. (1993). Inhibition of human immunodeficiency virus type 1 replication by hydroxychloroquine in T cells and monocytes. AIDS Res. Hum. Retrov..

[16] Chiang G., Sassaroli M., Louie M., Chen H., Stecher V.J., Sperber K. (1996). Inhibition of HIV-1 replication by hydroxychloroquine: Mechanism of action and comparison with zidovudine. Clin. Ther..

[17] Sperber K., Louie M., Kraus T., Proner J., Sapira E., Lin S., Stecher V., Mayer L. (1995). Hydroxychloroquine treatment of patients with human immunodeficiency virus type 1. Clin. Ther..

[18] Sperber K., Chiang G., Chen H., Ross W., Chusid E., Gonchar M., Chow R., Liriano O. (1997). Comparison of hydroxychloroquine with zidovudine in asymptomatic patients infected with human immunodeficiency virus type 1. Clin Ther.

[19] Paton N.I., Aboulhab J., Karim F. (2002). Hydroxychloroquine, hydroxycarbamide, and didanosine as economic treatment for HIV-1. The Lancet.

[20] Paton N., Aboulhab J.J. (2005). Hydroxychloroquine, hydroxyurea and didanosine as initial therapy for HIV-infected patients with low viral load: Safety, efficacy and resistance profile after 144 weeks. HIV Medicine.

[21] Aguirre-Cruz L., Torres K.J., Jung-Cook H., Fortuny C., Sánchez E., Soda-Mehry A., Sotelo J., Reyes-Terán G. (2010). Preferential concentration of hydroxychloroquine in adenoid tissue of HIV-infected subjects. AIDS Res. Hum. Retrov..

[22] González-Hernández I., Aguirre-Cruz L., Sotelo J., López-Arellano R., Morales-Hipólito A., Jung-Cook H. (2014). Distribution of hydroxychloroquine in lymphoid tissue in a rabbit model for HIV infection. Antimicrob. Agents Chemother..

[23] Piconi S., Parisotto S., Rizzardini G., Passerini S., Terzi R., Argenteri B., Meraviglia P., Capetti A., Biasin M., Trabattoni D. (2011). Hydroxychloroquine drastically reduces immune activation in HIV-infected, antiretroviral therapy–treated immunologic nonresponders. Blood.

[24] Paton N.I., Goodall R.L., Dunn D.T., Franzen S., Collaco-Moraes Y., Gazzard B.G., Williams I.G., Fisher M.J., Winston A., Fox J. (2012). Effects of hydroxychloroquine on immune activation and disease progression among HIV-infected patients not receiving antiretroviral therapy: A randomized controlled trial. JAMA Ophthalmol..

[25] Chen Y., Traore Y.L., Yang S., Lajoie J., Fowke K.R., Rickey D.W., Ho E.A. (2018). Implant delivering hydroxychloroquine attenuates vaginal T lymphocyte activation and inflammation. J. Control. Release.

[26] Padmakumar B., Jayan J.B., Menon R.M., Krishnankutty B., Payippallil R., Nisha R. (2009). Comparative evaluation of four therapeutic regimes in chikungunya arthritis: A prospective randomized parallel-group study. Indian J. Rheumatol..

[27] Bouquillard E., Fianu A., Bangil M., Charlette N., Ribéra A., Michault A., Favier F., Simon F., Flipo R.-M. (2018). Rheumatic manifestations associated with Chikungunya virus infection: A study of 307 patients with 32-month follow-up (RHUMATOCHIK study). Joint Bone Spine..

[28] Ravindran V., Alias G. (2017). Efficacy of combination DMARD therapy *vs*. hydroxychloroquine monotherapy in chronic persistent chikungunya arthritis: A 24-week randomized controlled open label study. Clin. Rheumatol..

[29] Pandya S. (2008). Methotrexate and hydroxychloroquine combination therapy in chronic chikungunya arthritis: A 16 week study. Indian J. Rheumatol..

[30] Helal G.K., Gad M.A., Abd-Ellah M.F., Eid M.S. (2016). Hydroxychloroquine augments early virological response to pegylated interferon plus ribavirin in genotype-4 chronic hepatitis C patients. J. Med. Virol..

[31] Cao B., Parnell L.A., Diamond M.S., Mysorekar I.U. (2017). Inhibition of autophagy limits vertical transmission of Zika virus in pregnant mice. J. Exp. Med..

[32] Gautret P., Lagier J.-C., Parola P., Meddeb L., Mailhe M., Doudier B., Courjon J., Giordanengo V., Vieira V.E., Dupont H.T. (2020). Hydroxychloroquine and azithromycin as a treatment of COVID-19: Results of an open-label non-randomized clinical trial. Int. J. Antimicrob. Agents.

[33] Gautret P., Lagier J.-C., Parola P., Meddeb L., Sevestre J., Mailhe M., Doudier B., Aubry C., Amrane S., Seng P. (2020). Clinical and microbiological effect of a combination of hydroxychloroquine and azithromycin in 80 COVID-19 patients with at least a six-day follow up: A pilot observational study. Travel Med. Infect. Di..

[34] Molina J.M., Delaugerre C., Goff J.L., Mela-Lima B., Ponscarme D., Goldwirt L., de Castro N. (2020). No evidence of rapid antiviral clearance or clinical benefit with the combination of hydroxychloroquine and azithromycin in patients with severe COVID-19 infection. Med. Mal. Infect..

[35] Tang W., Cao Z., Han M., Wang Z., Chen J., Sun W., Wu Y., Xiao W., Liu S., Chen E. (2020). Hydroxychloroquine in patients with COVID-19: An open-label, randomized, controlled trial. MedRxiv.

[36] Abd-Elsalam S., Esmail E.S., Khalaf M., Abdo E.F., Medhat M.A., Abd El Ghafar M.S., Ahmed O.A., Soliman S., Serangawy G.N., Alboraie M. (2020). Hydroxychloroquine in the treatment of COVID-19: A multicenter randomized controlled study. Am. J. Trop. Med. Hyg..

[37] Skipper C.P., Pastick K.A., Engen N.W., Bangdiwala A.S., Abassi M., Lofgren S.M., Williams D.A., Okafor E.C., Pullen M.F., Nicol M.R. (2020). Hydroxychloroquine in nonhospitalized adults with early COVID-19: A randomized trial. Ann. Intern. Med..

[38] Mahevas M., Tran V.-T., Roumier M., Chabrol A., Paule R., Guillaud C., Gallien S., Lepeule R., Szwebel T.-A., Lescure X. (2020). No evidence of clinical efficacy of hydroxychloroquine in patients hospitalized for COVID-19 infection with oxygen requirement: Results of a study using routinely collected data to emulate a target trial. MedRxiv.

[39] Mahévas M., Tran V.T., Roumier M., Chabrol A., Paule R., Guillaud C., Fois E., Lepeule R., Szwebel T.A., Lescure F.X. (2020). Clinical efficacy of hydroxychloroquine in patients with covid-19 pneumonia who require oxygen: Observational comparative study using routine care data. BMJ.

[40] Lee S.H., Son H., Peck K.R. (2020). Can post-exposure prophylaxis for COVID-19 be considered as one of outbreak response strategies in long-term care hospitals?. Int. J. Antimicrob. Agents.

[41] Boulware D.R., Pullen M.F., Bangdiwala A.S., Pastick K.A., Lofgren S.M., Okafor E.C., Skipper C.P., Nascene A.A., Nicol M.R., Abassi M. (2020). A randomized trial of hydroxychloroquine as postexposure prophylaxis for Covid-19. N. Engl. J. Med..

[42] French M.A., King M.S., Tschampa J.M., da Silva B.A., Landay A.L. (2009). Serum immune activation markers are persistently increased in patients with HIV infection after 6 years of antiretroviral therapy despite suppression of viral replication and reconstitution of CD4^+^ T cells. J. Infect. Dis..

[43] Ornstein M.H., Sperber K. (1996). The antiinflammatory and antiviral effects of hydroxychloroquine in two patients with acquired immunodeficiency syndrome and active inflammatory arthritis. Arthritis Rheum..

[44] Chaaithanya I.K., Muruganandam N., Sundaram S.G., Kawalekar O., Sugunan A.P., Manimunda S.P., Ghosal S.R., Muthumani K., Vijayachari P. (2011). Role of proinflammatory cytokines and chemokines in chronic arthropathy in CHIKV infection. Viral Immunol..

[45] Lin K.-M., Cheng T.-T., Lin J.-C., Chen C.-J. (2015). Tumor necrosis factor-α antagonist therapy for concomitant rheumatoid arthritis and hepatitis C virus infection: A case series study. Clin. Rheumatol..

[46] Marzano A., Angelucci E., Andreone P., Brunetto M., Bruno R., Burra P., Caraceni P., Daniele B., Di Marco V., Fabrizi F.J. (2007). Prophylaxis and treatment of hepatitis B in immunocompromised patients. Dig. Liver Dis..

[47] Zingarelli S., Airò P., Frassi M., Bazzani C., Scarsi M., Puoti M. (2008). Prophylaxis and therapy of HBV infection in 20 patients treated with disease modifying antirheumatic drugs or with biological agents for rheumatic diseases. Reumatismo.

[48] Mo Y.-Q., Liang A.-Q., Ma J.-D., Chen L.-F., Zheng D.-H., Schumacher H.R., Dai L. (2014). Discontinuation of antiviral prophylaxis correlates with high prevalence of hepatitis B virus (HBV) reactivation in rheumatoid arthritis patients with HBV carrier state: A real-world clinical practice. BMC Musculoskelet. Disord..

[49] World Health Organization Coronavirus Disease (COVID-19) Outbreak Situation. https://www.who.int/emergencies/diseases/novel-coronavirus-2019.

[50] Yao X., Ye F., Zhang M., Cui C., Huang B., Niu P., Liu X., Zhao L., Dong E., Song C. (2020). In vitro antiviral activity and projection of optimized dosing design of hydroxychloroquine for the treatment of severe acute respiratory syndrome coronavirus 2 (SARS-CoV-2). Clin. Infect. Dis..

[51] Million M., Lagier J.-C., Gautret P., Colson P., Fournier P.-E., Amrane S., Hocquart M., Mailhe M., Esteves-Vieira V., Doudier B. (2020). Full-length title: Early treatment of COVID-19 patients with hydroxychloroquine and azithromycin: A retrospective analysis of 1061 cases in Marseille, France. Travel Med. Infect. Di..

[52] Chorin E., Dai M., Shulman E., Wadhwani L., Bar-Cohen R., Barbhaiya C., Aizer A., Holmes D., Bernstein S., Spinelli M. (2020). The QT interval in patients with COVID-19 treated with hydroxychloroquine and azithromycin. Nat. Med..

[53] Chorin E., Wadhwani L., Magnani S., Dai M., Shulman E., Nadeau-Routhier C., Knotts R., Bar-Cohen R., Kogan E., Barbhaiya C. (2020). QT interval prolongation and torsade de pointes in patients with COVID-19 treated with hydroxychloroquine/azithromycin. Heart Rhythm.

[54] Mitra R.L., Greenstein S.A., Epstein L.M. (2020). An algorithm for managing QT prolongation in Coronavirus Disease 2019 (COVID-19) patients treated with either chloroquine or hydroxychloroquine in conjunction with azithromycin: Possible benefits of intravenous lidocaine. HeartRhythm Case Rep..

[55] Giudicessi J.R., Noseworthy P.A., Friedman P.A., Ackerman M.J. (2020). Urgent guidance for navigating and circumventing the QTc-prolonging and torsadogenic potential of possible pharmacotherapies for Coronavirus disease 19 (COVID-19). Mayo Clin. Proc..

[56] Liu J., Cao R., Xu M., Wang X., Zhang H., Hu H., Li Y., Hu Z., Zhong W., Wang M. (2020). Hydroxychloroquine, a less toxic derivative of chloroquine, is effective in inhibiting SARS-CoV-2 infection in vitro. Cell Discov..

[57] Kulkarni R.K., Kinikar A.A., Jadhav T. (2020). Adverse drug reaction profile of prophylactic hydroxychloroquine for COVID-19 among doctors. Med. J. DY Patil Vidyapeeth.

[58] Maisonnasse P., Guedj J., Contreras V., Behillil S., Solas C., Marlin R., Naninck T., Pizzorno A., Lemaitre J., Gonçalves A. (2020). Hydroxychloroquine in the treatment and prophylaxis of SARS-CoV-2 infection in non-human primates. Res. Square.

[59] Lahouati M., Mériglier E., Martin L., Bouchet S., Desclaux A., Bonnet F. (2020). COVID-19 infection also occurs in patients taking hydroxychloroquine. J. Antimicrob. Chemother..

[60] Monti S., Balduzzi S., Delvino P., Bellis E., Quadrelli V.S., Montecucco C. (2020). Clinical course of COVID-19 in a series of patients with chronic arthritis treated with immunosuppressive targeted therapies. Ann. Rheum. Dis..

[61] EUA Hydroxychloroquine Sulfate Health Care Provider Fact Sheet. https://www.fda.gov/emergency-preparedness-and-response/mcm-legal-regulatory-and-policy-framework/emergency-use-authorization.

[62] Coronavirus (COVID-19) Update: FDA Revokes Emergency Use Authorization for Chloroquine and Hydroxychloroquine. https://www.fda.gov/news-events/press-announcements/coronavirus-COVID-19-update-fda-revokes-emergency-use-authorization-chloroquine-and.

[63] Alhazzani W., Møller M.H., Arabi Y.M., Loeb M., Gong M.N., Fan E., Oczkowski S., Levy M.M., Derde L., Dzierba A. (2020). Surviving Sepsis Campaign: Guidelines on the management of critically ill adults with Coronavirus Disease 2019 (COVID-19). Intensive Care Med..

[64] Qaseem A., Yost J., Etxeandia-Ikobaltzeta I., Miller M.C., Abraham G.M., Obley A.J., Forciea M.A., Jokela J.A., Humphrey L.L. (2020). Should clinicians use chloroquine or hydroxychloroquine alone or in combination with azithromycin for the prophylaxis or treatment of COVID-19? Living practice points from the American college of physicians (Version 1). Ann. Intern. Med..

[65] Treatments for COVID-19: Canadian Arm of the SOLIDARITY Trial (CATCO). https://clinicaltrials.gov/ct2/show/NCT04330690.

[66] Randomised Evaluation of COVID-19 Therapy (RECOVERY). https://clinicaltrials.gov/ct2/show/NCT04381936.

[67] Trial of Treatments for COVID-19 in Hospitalized Adults (DisCoVeRy). https://clinicaltrials.gov/ct2/show/NCT04315948.

[68] World Health Organisation Q&A: Hydroxychloroquine and COVID-19. https://www.who.int/news-room/q-a-detail/q-a-hydroxychloroquine-and-covid-19.

[69] Kashour T., Tleyjeh I.M. (2020). It is time to drop hydroxychloroquine from our COVID-19 armamentarium. Med. Hypotheses.

[70] Cutler D. (1993). Possible mechanisms of action of antimalarials in rheumatic disease. Agents Actions Suppl..

[71] Garcia-Cremades M., Solans B.P., Hughes E., Ernest J.P., Wallender E., Aweeka F., Luetkemeyer A.F., Savic R.M. (2020). Optimizing hydroxychloroquine dosing for patients with COVID-19: An integrative modeling approach for effective drug repurposing. Clin. Pharmacol. Ther..

[72] Naso J.R., Wong D., Wong D.R., Chen C.-H., Hoang L.M. (2019). Tropheryma whipplei endocarditis presenting as chronic valvular disease: A case report and review of literature. Hum. Phatol. Case Rep..

[73] Vogl D.T., Stadtmauer E.A., Tan K.S., Heitjan D.F., Davis L.E., Pontiggia L., Rangwala R., Piao S., Chang Y.C., Scott E.C. (2014). Combined autophagy and proteasome inhibition: A phase 1 trial of hydroxychloroquine and bortezomib in patients with relapsed/refractory myeloma. Autophagy.

[74] Kanvinde S., Chhonker Y.S., Ahmad R., Yu F., Sleightholm R., Tang W., Jaramillo L., Chen Y., Sheinin Y., Li J. (2018). Pharmacokinetics and efficacy of orally administered polymeric chloroquine as macromolecular drug in the treatment of inflammatory bowel disease. Acta Biomater..

[75] Jancinova V., Pazourekova S., Lucova M., Perecko T., Mihalova D., Bauerova K., Nosal R., Drabikova K. (2015). Selective inhibition of extracellular oxidants liberated from human neutrophils-A new mechanism potentially involved in the anti-inflammatory activity of hydroxychloroquine. Int Immunopharmacol.

[76] The HERA Study Group (1995). A-randomized-trial-of-hydroxychloroquine-in-early-rheumatoid. Am. J. Med..

[77] Nuver-Zwart I., Van Riel P., Van de Putte L., Gribnau F. (1989). A double blind comparative study of sulphasalazine and hydroxychloroquine in rheumatoid arthritis: Evidence of an earlier effect of sulphasalazine. Ann. Rheum. Dis..

[78] Van Der Heijde D., Pl V.R. (1989). Effects of hydroxychloroquine and sulfasalazine on progression of joint damagein RA. Lancet.

[79] Batun-Garrido J.A.J., Salas-Magana M., Juarez-Rojop I.E. (2018). Association between leptin and IL-6 concentrations with cardiovascular risk in patients with rheumatoid arthritis. Clin. Rheumatol..

[80] Morris S.J., Wasko M.C., Antohe J.L., Sartorius J.A., Kirchner H.L., Dancea S., Bili A. (2011). Hydroxychloroquine use associated with improvement in lipid profiles in rheumatoid arthritis patients. Arthritis Care Res. (Hoboken).

[81] Solomon D.H., Garg R., Lu B., Todd D.J., Mercer E., Norton T., Massarotti E. (2014). Effect of hydroxychloroquine on insulin sensitivity and lipid parameters in rheumatoid arthritis patients without diabetes mellitus: A randomized, blinded crossover trial. Arthritis Care Res. (Hoboken).

[82] Penn S.K., Kao A.H., Schott L.L., Elliott J.R., Toledo F.G., Kuller L., Manzi S., Wasko M.C. (2010). Hydroxychloroquine and glycemia in women with rheumatoid arthritis and systemic lupus erythematosus. J. Rheumatol.

[83] The Canadian Hydroxychloroquine Study Group T. (1991). A randomized study of the effect of withdrawing hydroxychloroquine sulfate in systemic lupus erythematosus. N. Engl. J. Med..

[84] Tsakonas E., Joseph L., Esdaile J.M., Choquette D., Senecal J.L., Cividino A., Danoff D., Osterland C.K., Yeadon C. (1998). A long-term study of hydroxychloroquine withdrawal on exacerbations in systemic lupus erythematosus. The Canadian Hydroxychloroquine Study Group. Lupus.

[85] Alarcon G.S., McGwin G., Bertoli A.M., Fessler B.J., Calvo-Alen J., Bastian H.M., Vila L.M., Reveille J.D., Group L.S. (2007). Effect of hydroxychloroquine on the survival of patients with systemic lupus erythematosus: Data from LUMINA, a multiethnic US cohort (LUMINA L). Ann. Rheum. Dis..

[86] An N., Chen Y., Wang C., Yang C., Wu Z.H., Xue J., Ye L., Wang S., Liu H.F., Pan Q. (2017). Chloroquine autophagic nhibition rebalances Th17/Treg-mediated immunity and ameliorates systemic Lupus erythematosus. Cell. Physiol. Biochem..

[87] Monzavi S.M., Alirezaei A., Shariati-Sarabi Z., Tavakol Afshari J., Mahmoudi M., Dormanesh B., Jahandoost F., Khoshdel A.R., Etemad Rezaie A. (2018). Efficacy analysis of hydroxychloroquine therapy in systemic Lupus erythematosus: A study on disease activity and immunological biomarkers. Inflammopharmacology.

[88] Willis R., Seif A., McGwin Jr G., Martinez-Martinez L., Gonzalez E., Dang N., Papalardo E., Liu J., Vilá L., Reveille J. (2012). Effect of hydroxychloroquine treatment on pro-inflammatory cytokines and disease activity in SLE patients: Data from LUMINA (LXXV), a multiethnic US cohort. Lupus.

[89] Shimomatsu T., Li H., Ikeda T., Kanazawa N., Furukawa F. The effect of hydroxychloroquine on the lupus erythematosus-like skin lesions in MRL/Ipr mice. Proceedings of the International Investigative Dermatology.

[90] Gomez-Guzman M., Jimenez R., Romero M., Sanchez M., Zarzuelo M.J., Gomez-Morales M., O’Valle F., Lopez-Farre A.J., Algieri F., Galvez J. (2014). Chronic hydroxychloroquine improves endothelial dysfunction and protects kidney in a mouse model of systemic lupus erythematosus. Hypertension.

[91] Virdis A., Tani C., Duranti E., Vagnani S., Carli L., Kuhl A.A., Solini A., Baldini C., Talarico R., Bombardieri S. (2015). Early treatment with hydroxychloroquine prevents the development of endothelial dysfunction in a murine model of systemic lupus erythematosus. Arthritis Res. Ther..

[92] Wallace D.J., Metzger A.L., Stecher V.J., Turnbull B.A., Kern P.A. (1990). Cholesterol-lowering effect of hydroxychloroquine in patients with rheumatic disease reversal of deleterious effects of steroids on lipids. Am. J. Med..

[93] Levy R., Vilela V., Cataldo M., Ramos R., Duarte J.L., Tura B., Albuquerque E.M., Jesus N. (2001). Hydroxychloroquine (HCQ) in lupus pregnancy double-blind and placebo-controlled study. Lupus.

[94] Clowse M.E., Magder L., Witter F., Petri M. (2006). Hydroxychloroquine in lupus pregnancy. Arthritis Rheum..

[95] Bertolaccini M.L., Contento G., Lennen R., Sanna G., Blower P.J., Ma M.T., Sunassee K., Girardi G. (2016). Complement inhibition by hydroxychloroquine prevents placental and fetal brain abnormalities in antiphospholipid syndrome. J. Autoimmun.

[96] Syang Pyng Gan M. (2016). Swee Gaik Ong, FRCP, Antithrombotic effects of hydroxychloroquine in a pregnant patient with antiphospholipid syndrome and recurrent venous thromboembolism. Med. J. Malaysia.

[97] Higgins J.P., Thomas J., Chandler J., Cumpston M., Li T., Page M.J., Welch V.A. (2019). Cochrane Handbook for Systematic Reviews of Interventions.

[98] Urbanski G., Caillon A., Poli C., Kauffenstein G., Begorre M.A., Loufrani L., Henrion D., Belizna C. (2018). Hydroxychloroquine partially prevents endothelial dysfunction induced by anti-beta-2-GPI antibodies in an in vivo mouse model of antiphospholipid syndrome. PLoS ONE.

[99] Schreiber K., Breen K., Parmar K., Rand J.H., Wu X.X., Hunt B.J. (2018). The effect of hydroxychloroquine on haemostasis, complement, inflammation and angiogenesis in patients with antiphospholipid antibodies. Rheumatology.

[100] Fox R.I., Chan E., Benton L., Fong S., Friedlaender M., Howell F.V. (1988). Treatment of primary Sjögren’s syndrome with hydroxychloroquine. Am. J. Med..

[101] Cankaya H., Alpoz E., Karabulut G., Guneri P., Boyacioglu H., Kabasakal Y. (2010). Effects of hydroxychloroquine on salivary flow rates and oral complaints of Sjogren patients: A prospective sample study. Oral Surg. Oral Med. Oral Pathol. Oral Radiol. Endodontol..

[102] Yavuz S., Asfuroglu E., Bicakcigil M., Toker E. (2011). Hydroxychloroquine improves dry eye symptoms of patients with primary Sjogren’s syndrome. Rheumatol Int.

[103] Li C.L., He J., Li Z.G., Zheng L.W., Hua H. (2013). Effects of total glucosides of paeony for delaying onset of Sjogren’s syndrome: An animal study. J. Craniomaxillofac Surg.

[104] Wu G., Pu X., Yu G., Li T. (2015). Effects of total glucosides of peony on AQP-5 and its mRNA expression in submandibular glands of NOD mice with Sjogren’s syndrome. Eur. Rev. Med. Pharmacol. Sci..

[105] Emami J., Gerstein H.C., Pasutto F.M., Jamali F. (1999). Insulin-sparing effect of hydroxychloroquine in diabetic rats is concentration dependent. Can. J. Physiol. Pharmacol..

[106] Abdel-Hamid A.A., El-Firgany Ael D. (2016). Hydroxychloroquine hindering of diabetic isletopathy carries its signature on the inflammatory cytokines. J. Mol. Histol..

[107] Gerstein H.C., Thorpe K.E., Haynes R.B., Taylor D.W. (2002). Gerstein_The effectiveness of hydroxychloroquine in patients with type 2 diabetes mellitus who are refractory to sulfonylureas a randomized trial. Diabetes Res. Clin. Pract..

[108] Mercer E., Rekedal L., Garg R., Lu B., Massarotti E.M., Solomon D.H. (2012). Hydroxychloroquine improves insulin sensitivity in obese non-diabetic individuals. Arthritis Res. Ther..

[109] Sheikhbahaie F., Amini M., Gharipour M., Aminoroaya A., Taheri N. (2016). The effect of hydroxychloroquine on glucose control and insulin resistance in the prediabetes condition. Adv. Biomed. Res..

[110] Pareek A., Chandurkar N., Thomas N., Viswanathan V., Deshpande A., Gupta O.P., Shah A., Kakrani A., Bhandari S., Thulasidharan N.K. (2014). Efficacy and safety of hydroxychloroquine in the treatment of type 2 diabetes mellitus: A double blind, randomized comparison with pioglitazone. Curr. Med. Res. Opin..

[111] Yao J., Xie J., Xie B., Li Y., Jiang L., Sui X., Zhou X., Pan H., Han W. (2016). Therapeutic effect of hydroxychloroquine on colorectal carcinogenesis in experimental murine colitis. Biochem. Pharmacol..

[112] Bourke L., McCormick J., Taylor V., Pericleous C., Blanchet B., Costedoat-Chalumeau N., Stuckey D., Lythgoe M.F., Stephanou A., Ioannou Y. (2015). Hydroxychloroquine Protects against Cardiac Ischaemia/Reperfusion Injury In Vivo via Enhancement of ERK1/2 Phosphorylation. PLoS ONE.

[113] Abdel-Hamid A.A.M., Firgany A.E.L. (2016). Favorable outcomes of hydroxychloroquine in insulin resistance may be accomplished by adjustment of the endothelial dysfunction as well as the skewed balance of adipokines. Acta Histochem..

[114] Shukla A.M., Bose C., Karaduta O.K., Apostolov E.O., Kaushal G.P., Fahmi T., Segal M.S., Shah S.V. (2015). Impact of hydroxychloroquine on atherosclerosis and vascular stiffness in the presence of chronic kidney disease. PLoS ONE.

[115] Long L., Yang X., Southwood M., Lu J., Marciniak S.J., Dunmore B.J., Morrell N.W. (2013). Chloroquine prevents progression of experimental pulmonary hypertension via inhibition of autophagy and lysosomal bone morphogenetic protein type II receptor degradation. Circ. Res..

[116] Ruiz A., Rockfield S., Taran N., Haller E., Engelman R.W., Flores I., Panina-Bordignon P., Nanjundan M. (2016). Effect of hydroxychloroquine and characterization of autophagy in a mouse model of endometriosis. Cell Death Dis.

[117] Firestein G.S., McInnes I.B. (2017). Immunopathogenesis of rheumatoid arthritis. Immunity.

[118] McInnes I.B., Schett G. (2017). Pathogenetic insights from the treatment of rheumatoid arthritis. The Lancet.

[119] Tam L., LiU E., Lam C., Tomlinsoni B. (2000). Hydroxychloroquine has no significant effect on lipids and apolipoproteins in Chinese systemic lupus erythematosus patients with mild or inactive disease. Lupus.

[120] Moroni G., Doria A., Giglio E., Tani C., Zen M., Strigini F., Zaina B., Tincani A., de Liso F., Matinato C. (2016). Fetal outcome and recommendations of pregnancies in lupus nephritis in the 21st century. A prospective multicenter study. J. Autoimmun..

[121] Kruize A.A., Hene R., Kallenberg C.G., van Bijsterveld P., van der Heide A., Kater L., Bijlsma J. (1993). Hydroxychloroquine treatment for primary Sjögren’s syndrome a two year double blind crossover trial. Ann. Rheum. Dis..

[122] Raoult D., Marrie T. (1995). Q fever. Clin. Infect. Dis..

[123] Fenollar F., Fournier P.-E., Carrieri M.P., Habib G., Messana T., Raoult D. (2001). Risks factors and prevention of Q fever endocarditis. Clin. Infect. Dis..

[124] Kampschreur L.M., Dekker S., Hagenaars J.C., Lestrade P.J., Renders N.H., de Jager-Leclercq M.G., Hermans M.H., Groot C.A., Groenwold R.H., Hoepelman A.I. (2012). Identification of risk factors for chronic Q fever, the Netherlands. Emerg. Infect. Dis..

[125] Smith C.B., Evavold C., Kersh G.J. (2019). The Effect of pH on Antibiotic Efficacy against Coxiella burnetii in Axenic Media. Sci. Rep..

[126] Maurin M., Benoliel A.M., Bongrand P., Raoult D. (1992). Phagolysosomal alkalinization and the bactericidal effect of antibiotics: The Coxiella burnetii paradigm. J. Infect. Dis..

[127] Brouqui P.R.D. (1993). Treatment of Q fever endocarditis by doxycycline and hydroxychloroquine. Clin. Infect. Dis..

[128] Lupoglazoff J., Brouqui P., Magnier S., Hvass U., Casasoprana A. (1997). Q fever tricuspid valve endocarditis. Arch. Dis. Child..

[129] Raoult D., Houpikian P., Tissot Dupont H., Riss J.M., Arditi-Djiane J., Brouqui P. (1999). Treatment of Q fever endocarditis: Comparison of 2 regimens containing doxycycline and ofloxacin or hydroxychloroquine. Arch. Intern. Med..

[130] Elzein F.E., Alsherbeeni N., Alnajashi K., Alsufyani E., Akhtar M., Albalawi R., Albarrag A.M., Kaabia N., Mehdi S., Alzahrani A. (2019). Ten-year experience of Q fever endocarditis in a tertiary cardiac center in Saudi Arabia. Int. J. Infect. Dis..

[131] Gonçalves M., Moreira S., Gaspar E., Santos L. (2018). Rare case of otomastoiditis due to *Coxiella burnetii* chronic infection. Case Rep..

[132] Allan-Blitz L.-T., Sakona A., Wallace W.D., Klausner J.D. (2018). *Coxiella burnetii* endocarditis and meningitis, California, USA, 2017. Emerg. Infect. Dis..

[133] Jalal Z., Duperril M., Séguéla P.-E., Melenotte C., Chabaneix J., Raoult D., Thambo J.-B. (2018). First case of Q fever endocarditis involving the Melody^®^ transcatheter pulmonary valve in an Afebrile child. Pediatr. Cardiol..

[134] Stokes W., Janvier J., Vaughan S., Microbiology M. (2016). Chronic Q fever in Alberta: A case of Coxiella burnetii mycotic aneurysm and concomitant vertebral osteomyelitis. Can. J. Infect. Dis. Med..

[135] Polo M., Mastrandrea S., Santoru L., Arcadu A., Masala G., Marras V., Bagella G., Sechi M., Tanda F., Pirina P.J. (2015). Pulmonary inflammatory pseudotumor due to *Coxiella burnetii*. Case report and literature review. Microbes Infect..

[136] Salvia A.M., Cuviello F., Coluzzi S., Nuccorini R., Attolico I., Pascale S.P., Bisaccia F., Pizzuti M., Ostuni A. (2017). Expression of some ATP-binding cassette transporters in acute myeloid leukemia. Hematol. Rep..

[137] Miglionico R., Ostuni A., Armentano M.F., Milella L., Crescenzi E., Carmosino M., Bisaccia F. (2017). ABCC6 knockdown in HepG2 cells induces a senescent-like cell phenotype. Cell. Mol. Biol. Lett..

[138] Wang F., Zhang Z., Leung W.T., Chen J., Yi J., Ying C., Yuan M., Wang M., Zhang N., Qiu X. (2019). Hydroxychloroquine reverses the drug resistance of leukemic K562/ADM cells by inhibiting autophagy. Mol. Med. Rep..

[139] Godinho I., Nogueira E., Santos C., Paulo S., Fortes A., Guerra J., da Costa A.G. (2015). Chronic Q fever in a renal transplant recipient: A case report. Transplant Proc..

[140] Merhej V., Tattevin P., Revest M., Le Touvet B., Raoult D.J. (2012). Q fever osteomyelitis: A case report and literature review. Comp. Immunol. Microbiol. Infect..

[141] Million M., Walter G., Thuny F., Habib G., Raoult D. (2013). Evolution from acute Q fever to endocarditis is associated with underlying valvulopathy and age and can be prevented by prolonged antibiotic treatment. Clin. Infect. Dis..

[142] Million M., Thuny F., Richet H., Raoult D. (2010). Long-term outcome of Q fever endocarditis: A 26-year personal survey. Lancet Infect. Dis..

[143] Munster T., Gibbs J.P., Shen D., Baethge B.A., Botstein G.R., Caldwell J., Dietz F., Ettlinger R., Golden H.E., Lindsley H. (2002). Hydroxychloroquine concentration–response relationships in patients with rheumatoid arthritis. Arthritis Rheum. Off. J. Am. Coll. Rheumatol..

[144] Drucker A.M., Rosen C.F. (2011). Drug-induced photosensitivity. Drug. Saf..

[145] Angelakis E., Million M., Kankoe S., Lagier J.C., Armougom F., Giorgi R., Raoult D. (2014). Abnormal weight gain and gut microbiota modifications are side effects of long-term doxycycline and hydroxychloroquine treatment. Antimicrob. Agents Chemother..

[146] Anderson A., Bijlmer H., Fournier P.-E., Graves S., Hartzell J., Kersh G.J., Limonard G., Marrie T.J., Massung R.F., McQuiston J.H. (2013). Diagnosis and management of Q fever—United States, 2013: Recommendations from CDC and the Q fever working group. Morb. Mortal. Wkly. Rep..

[147] Fenollar F., Puéchal X., Raoult D. (2007). Whipple’s disease. N. Engl. J. Med..

[148] Lagier J.-C., Lepidi H., Raoult D., Fenollar F. (2010). Systemic *Tropheryma whipplei*: Clinical presentation of 142 patients with infections diagnosed or confirmed in a reference center. Medicine.

[149] Lagier J.-C., Fenollar F., Lepidi H., Raoult D. (2010). Failure and relapse after treatment with trimethoprim/sulfamethoxazole in classic Whipple’s disease. J. Antimicrob. Chemoth..

[150] Emonet S., Wuillemin T., Harbarth S., Wassilew N., Cikirikcioglu M., Schrenzel J., Lagier J.-C., Raoult D., Van Delden C. (2015). Relapse of Tropheryma whipplei endocarditis treated by trimethoprim/sulfamethoxazole, cured by hydroxychloroquine plus doxycycline. Int. J. Infect. Dis..

[151] Boulos A., Rolain J., Mallet M., Raoult D. (2005). Molecular evaluation of antibiotic susceptibility of Tropheryma whipplei in axenic medium. J. Antimicrob. Chemother..

[152] Bakkali N., Fenollar F., Biswas S., Rolain J.-M., Raoult D. (2008). Acquired resistance to trimethoprim-sulfamethoxazole during Whipple disease and expression of the causative target gene. J. Infect. Dis..

[153] Fenollar F., Rolain J.-M., Alric L., Papo T., Chauveheid M.-P., van de Beek D., Raoult D. (2009). Resistance to trimethoprim/sulfamethoxazole and Tropheryma whipplei. Int. J. Antimicrob. Agents.

[154] Boulos A., Rolain J.-M., Raoult D. (2004). Antibiotic susceptibility of *Tropheryma whipplei* in MRC5 cells. Antimicrob. Agents Chemother..

[155] Lagier J.-C., Fenollar F., Lepidi H., Giorgi R., Million M., Raoult D. (2014). Treatment of classic Whipple’s disease: From in vitro results to clinical outcome. J. Antimicrob. Chemoth..

[156] Le Goff M., Cornec D., Guellec D., Marhadour T., Devauchelle-Pensec V., Jousse-Joulin S., Herbette M., Cauvin J.M., Le Guillou C., Renaudineau Y. (2019). Peripheral-blood b-cell subset disturbances in inflammatory joint diseases induced by Tropheryma whipplei. PLoS ONE.

[157] Brönnimann D., Vareil M.-O., Sibon I., Lagier J.-C., Lepidi H., Puges M., Haneche F., Raoult D., Desclaux A., Neau D. (2019). Limbic encephalitis as a relapse of Whipple’s disease with digestive involvement and spondylodiscitis. Infection.

[158] Gaudé M., Tébib J., Puéchal X. (2015). Atypical focal forms of Whipple’s disease seen by rheumatologists. Joint Bone Spine.

[159] Lenfant M., Callemeyn J., Alaerts H., Meersseman W., Van W.M. (2019). Whipple’s disease in a man of North African descent: Case report and brief review of the literature. Acta Gastroenterol. Belg..

[160] Vayssade M., Tournadre A., D’Incan M., Soubrier M., Dubost J.-J. (2015). Immune reconstitution inflammatory syndrome during treatment of Whipple’s disease. Joint Bone Spine.

[161] Spoerl D., Bär D., Cooper J., Vogt T., Tyndall A., Walker U.A. (2009). Multisegmental spondylitis due to Tropheryma whipplei: Case report. Orphanet J. Rare Dis..

[162] De Jong P., Hazes J., Barendregt P., Huisman M., van Zeben D., Van Der Lubbe P., Gerards A., de Jager M., de Sonnaville P., Grillet B. (2013). Induction therapy with a combination of DMARDs is better than methotrexate monotherapy: First results of the tREACH trial. Ann. Rheum. Dis..

[163] Möttönen T., Hannonen P., Leirisalo-Repo M., Nissilä M., Kautiainen H., Korpela M., Laasonen L., Julkunen H., Luukkainen R., Vuori K. (1999). Comparison of combination therapy with single-drug therapy in early rheumatoid arthritis: A randomised trial. Lancet.

[164] Cobankara V., Ozatli D., Kiraz S., Ozturk M.A., Ertenli I., Turk T., Apras S., Haznedaroglu I.C., Calguneri M. (2004). Successful treatment of rheumatoid arthritis is associated with a reduction in serum sE-selectin and thrombomodulin level. Clin. Rheumatol..

[165] Kuriachan M., Revikumar K., Jolly A. (2012). Comparison of treatment outcome in rheumatoid arthritis patients treated with single and two DMARDs in combination with corticosteroids. Int. J. Drug Dev. Res..

[166] Roivainen A., Hautaniemi S., Mottonen T., Nuutila P., Oikonen V., Parkkola R., Pricop L., Ress R., Seneca N., Seppanen M. (2013). Correlation of 18F-FDG PET/CT assessments with disease activity and markers of inflammation in patients with early rheumatoid arthritis following the initiation of combination therapy with triple oral antirheumatic drugs. Eur J. Nucl Med. Mol Imaging.

[167] Tynjala P., Vahasalo P., Tarkiainen M., Kroger L., Aalto K., Malin M., Putto-Laurila A., Honkanen V., Lahdenne P. (2011). Aggressive combination drug therapy in very early polyarticular juvenile idiopathic arthritis (ACUTE-JIA): A multicentre randomised open-label clinical trial. Ann. Rheum. Dis..

[168] Chafin C.B., Regna N.L., Hammond S.E., Reilly C.M. (2013). Cellular and urinary microRNA alterations in NZB/W mice with hydroxychloroquine or prednisone treatment. Int. Immunopharmacol..

[169] Fasano S., Pierro L., Pantano I., Iudici M., Valentini G. (2017). Longterm hydroxychloroquine therapy and low-dose aspirin may have an additive effectiveness in the primary prevention of cardiovascular events in patients with systemic Lupus erythematosus. J. Rheumatol..

[170] Schultz K., Gilman A.L. (1997). Immune suppression by lysosomotropic amines and cyclosporine on T-cell responses to minor and major histocompatibility antigens. Leukemia Lymphoma.

[171] Faraone I., Sinisgalli C., Ostuni A., Armentano M.F., Carmosino M., Milella L., Russo D., Labanca F., Khan H. (2020). Astaxanthin anticancer effects are mediated through multiple molecular mechanisms: A systematic review. Pharmacol. Res..

[172] Hooijmans C.R., Rovers M.M., De Vries R.B., Leenaars M., Ritskes-Hoitinga M., Langendam M.W. (2014). SYRCLE’s risk of bias tool for animal studies. BMC Med. Res. Methodol..

